# Targeting S100A9 protein affects mTOR-ER stress signaling and increases venetoclax sensitivity in Acute Myeloid Leukemia

**DOI:** 10.1038/s41408-023-00962-z

**Published:** 2023-12-18

**Authors:** Rong Fan, Hatice Satilmis, Niels Vandewalle, Emma Verheye, Elke De Bruyne, Eline Menu, Nathan De Beule, Ann De Becker, Gamze Ates, Ann Massie, Tessa Kerre, Marie Törngren, Helena Eriksson, Karin Vanderkerken, Karine Breckpot, Ken Maes, Kim De Veirman

**Affiliations:** 1 Laboratory for Hematology and Immunology, Department of Biomedical Sciences, Vrije Universiteit Brussel (VUB), Laarbeeklaan 103, Building D, 1090 Brussel, Belgium; 2 Translational Oncology Research Center, Vrije Universiteit Brussel (VUB), Laarbeeklaan 103, Building D, 1090 Brussel, Belgium; 3 Laboratory of Myeloid Cell Immunology, VIB Center for Inflammation Research, Pleinlaan 2, 1050 Brussels, Belgium; 4 Department of Clinical Hematology, Universitair Ziekenhuis Brussel (UZ Brussel), Vrije Universiteit Brussel, Brussels, Belgium. Laarbeeklaan 101, 1090 Brussel, Belgium; 5 Neuro-Aging & Viro-Immunotherapy, Center for Neurosciences, Vrije Universiteit Brussel (VUB), Laarbeeklaan 103, 1090 Brussel, Belgium; 6 Department of Hematology, Ghent University Hospital, Faculty of Medicine and Health Sciences, Ghent University, 9000 Ghent, Belgium; 7 Active Biotech AB, Lund, Sweden. Scheelevägen 22, 22363 Lund, Sweden; 8 Laboratory for Molecular and Cellular Therapy, Department of Biomedical Sciences, Vrije Universiteit Brussel (VUB), Laarbeeklaan 103, 1090 Brussel, Belgium; 9 Clinical Sciences, Research Group Reproduction and Genetics, Centre for Medical Genetics, Vrije Universiteit Brussel (VUB), Universitair Ziekenhuis Brussel (UZ Brussel), Laarbeeklaan 103, 1090 Brussel, Belgium

**Keywords:** Targeted therapies, Cancer metabolism, Acute myeloid leukaemia

## Abstract

Acute Myeloid Leukemia (AML) is a heterogeneous disease with limited treatment options and a high demand for novel targeted therapies. Since myeloid-related protein S100A9 is abundantly expressed in AML, we aimed to unravel the therapeutic impact and underlying mechanisms of targeting both intracellular and extracellular S100A9 protein in AML cell lines and primary patient samples. S100A9 silencing in AML cell lines resulted in increased apoptosis and reduced AML cell viability and proliferation. These therapeutic effects were associated with a decrease in mTOR and endoplasmic reticulum stress signaling. Comparable results on AML cell proliferation and mTOR signaling could be observed using the clinically available S100A9 inhibitor tasquinimod. Interestingly, while siRNA-mediated targeting of S100A9 affected both extracellular acidification and mitochondrial metabolism, tasquinimod only affected the mitochondrial function of AML cells. Finally, we found that S100A9-targeting approaches could significantly increase venetoclax sensitivity in AML cells, which was associated with a downregulation of BCL-2 and c-MYC in the combination group compared to single agent therapy. This study identifies S100A9 as a novel molecular target to treat AML and supports the therapeutic evaluation of tasquinimod in venetoclax-based regimens for AML patients.

## Introduction

Acute Myeloid Leukemia (AML) is an aggressive hematological cancer characterized by the clonal expansion of myeloid progenitors (or blasts) in the bone marrow and blood [1]. The current treatment paradigm in fit AML patients employs remission-inducing chemotherapy with cytarabine- and anthracycline-containing regimens, followed by consolidation therapy to prolong remission, or allogeneic stem cell transplantation [2]. The combination of hypomethylating agent vidaza with BCL2-inhibitor venetoclax is the standard of care for unfit, elderly AML patients. The past years, novel targeted therapies including fms-like tyrosine kinase 3 (FLT3) inhibitors and isocitrate dehydrogenase 1 and 2 (IDH1/IDH2) inhibitors were incorporated into the therapeutic landscape of AML [3]. However, with a general 5-year overall survival rate of 29.5%, that even drops to 15% for AML patients aged 60 and older, the overall prognosis remains dismal [4].

The proinflammatory cytokine S100A9, and its binding partner S100A8, are calcium-binding proteins that form homodimers, heterodimers, and higher-order complexes that are essential for their biological functions [5, 6]. Once secreted, S100A9 can interact with receptors such as Toll-like receptor 4 (TLR4) and receptor for advanced glycation end-product (RAGE) [7, 8]. S100A9 is overexpressed in various human cancer types including breast cancer and non-small cell lung cancer, and this overexpression often correlates with a poor prognosis [9]. Within the tumor microenvironment, S100A9 is highly expressed by various myeloid cell populations (e.g., myeloid-derived suppressor cells) and plays a critical role in the formation of an immunosuppressive niche which favors tumor growth and drug resistance [10, 11].

Since S100A9 is a key regulator of myeloid differentiation and is highly expressed in AML cells, Laouedj et al. studied the impact of recombinant S100A9 protein on AML growth and survival. In vitro data revealed that recombinant S100A9 induced AML cell differentiation, whereas S100A8 protein maintained AML cells in an immature phenotype [12]. Intriguingly, recombinant S100A9 increased AML cell maturation, induced growth arrest and prolonged survival in an AML mouse model, indicative for anti-tumoral properties [13, 14]. Despite these interesting observations, the exact function of S100A9 (intracellular/extracellular) and the effect of S100A9-targeted therapies in AML cells remains to be elucidated.

In the present study, we evaluated the effect of S100A9 silencing and pharmacological inhibition of S100A9 in AML cells. Using RNA sequencing, Seahorse analysis, flow cytometry and western blot, we aimed to provide more mechanistic insights into the S100A9-mediated downstream effects in AML cells. As S100A9 was previously linked with venetoclax resistance in AML patients, we examined the effect of S100A9-targeting as potential sensitizer to venetoclax therapy in AML cell lines and primary patient samples.

## Material and methods

### Cell culture

The in vitro experiments were performed using human AML cell lines MOLM-13, KG-1a, OCI-AML3 and THP-1. KG-1a, MOLM-13, OCI-AML3 were maintained in RPMI-1640 medium (Gibco; Thermo Fisher Scientific, Inc., Waltham, MA, USA), THP-1 was cultured in RPMI-1640 medium supplemented with β-mercapto-ethanol (β-ME) and MV4-11 was cultured in IMDM at 37 °C in 5% CO_2_. All media were supplemented with 10% fetal bovine serum (FBS) (Biochrom AG, Berlin, Germany), 100 U/mL penicillin, 100 μg/mL streptomycin and 2 mM L-glutamine (Lonza, Basel, Switzerland). Cell lines were regularly tested for mycoplasma contamination and passaged no more than one month prior to experiments (Lonza, USA, #LT07-418). Frozen bone marrow mononuclear cells (BMMCs) or peripheral blood monocytes (PBMCs) from AML patients were thawed at 37 °C in RPMI-1640 medium containing 20% FBS, DNase I (100 μg/mL) and MgCl_2_ (1 μM). Sample collection and use of AML samples was approved by the medical ethics committee by Universitair Ziekenhuis Brussel (BUN: 1432021000659) and informed consent was obtained from all included patients.

### Compounds

Tasquinimod was purchased from Sigma-Aldrich (Diegem, Belgium) and dissolved in dimethylsulfoxide at a stock concentration of 5 mM. For the final concentration, we chose the concentrations 5, 10 and 25 µM. Venetoclax (ABT-199) was purchased from Selleck Chemicals (Houston, Texas, USA) and the final concentrations were 100, 250 and 500 nM for AML cells, and 10, 20 and 50 nM for primary AML samples.

### Small interfering (siRNA) transfection

S100A9 knockdown was achieved using specific siRNA from Qiagen (Antwerp, Belgium, #SI04317012) according to the manufacturer’s protocol. The gene-specific sequences were as follows: Hs_S100A9_10: TCG CAG CTG GAA CGC AAC ATA (selected for further experiments), Hs_S100A9_9: CAT CAA CAC CTT CCA CCA ATA, Hs_S100A9_7: CTC GGC TTT GAC AGA GTG CAA and Hs_S100A9_2: ATG GAG GAC CTG GAC ACA AAT. Briefly, 1.0 × 10^5^ cells/24-well or 5.0 × 10^5^ cells/6-well were seeded 1 h prior to transfection. Subsequently, cells were transfected with respectively 100 µl or 500 µl of Opti-MEM (Gibco) containing 20 nM siRNAs targeting S100A9, or negative control siRNA (scramble) (Qiagen, Catalog #1,027,280) using Lipofectamine 2000 (Thermo Fisher Scientific). The transfection efficiency was assessed by flow cytometry using a fluorescently labeled, non-targeting siRNA.

### Cell apoptosis, proliferation and cell cycle assays

AML cell lines (KG-1a, MOLM-13) were cultured at 1.0 × 10^5^ cells in 500 µL in the 24-well plate and were treated with siRNA for 72 h. Primary AML samples (1 PB, 4 BM) were cultured at 1.0 × 10^5^ cells in 200 µL in the round-bottom 96-well plate. For the combination experiments, AML cell lines were pre-treated with tasquinimod or S100A9-siRNA for 24 h and were afterwards incubated with venetoclax for an additional 24 h. To assess the effects of tasquinimod, cells were treated with increasing concentrations of the compound (10, 25 µM) for 24 h and 48 h. Apoptosis was measured by flow cytometry using Annexin-V and 7-aminoactinomycin D (7-AAD) (BD Biosciences, Erembodegem, Belgium) staining. Flow cytometry data were analyzed using BD FACSDiva Software (Becton Dickinson) and FlowJo software (Tree Star: Ashland, OR, USA).

To assess AML cell proliferation, 1 mg/mL bromodeoxyuridine (BrdU) (Sigma, #B5002) was added 4 h before sample collection. Samples were washed with FACS flow and fixed/incubated for 10 min with paraformaldehyde at 4 °C. The cells were incubated overnight in PBS (Gibco) 0.2% Tween (Sigma-Aldrich) at room temperature, washed twice with FACS flow, stained for 30 min with 2 M HCl and washed with both FACS flow and a mixture of PBS + 0.5% Triton X (Sigma-Aldrich) + 10% FBS. Cells were then stained with 3 µL of anti-BrdU-Fluorescein (Sigma-Aldrich, #112022693011) in 50 µL of PBS + 0.5% Triton X + 10% FBS and incubated in the dark for 30 min. After a washing step, a mixture of 200 µL FACS flow + 100 µL propidium iodide was added. The percentage of BrdU^+^ cells was detected by flow cytometry using the FACS Canto flow cytometer (BD Biosciences, Belgium).

### RNA sequencing and analysis

Total RNA was extracted and purified using the NucleoSpin RNA plus kit (Macherey–Nagel, Düren, Germany). Sample quality was checked by calculating the RNA integrity number (RIN value) using a fragment analyser (Agilent), with 3 biological replicates from each group. The RNA sequencing (RNA-seq) library preparation was performed with 150 ng RNA using the Illumina KAPA RNA HyperPrep kit with RiboErase (HMR) (Illumina, Cambridge, UK). Paired-end RNA-seq (2*100 bp) was done with an Illumina NovaSeq 6000 sequencing instrument (Illumina, Cambridge, UK) and read pairs were mapped to the human GRCh37 reference genome using the STAR alignment algorithm. All statistical analyses were performed with the statistics software R (version 4.2.2) and R packages obtained through the BioConductor project (https://www.bioconductor.org). The expression level of each gene was summarized and normalized using DESeq2 R/Bioconductor package and differential expression analysis was performed using DESeq2 pipeline. *P*-values were adjusted to control the global false discovery rate (FDR) across all comparisons with the default option of the DESeq2 package. Genes were considered differentially expressed if they had an adjusted *P*-value equal or lower than 0.05 and a fold change of more or equal to 2. Pathway enrichment analyses were performed using online curated gene set collection on the Gene Set Enrichment Analysis software (GSEA) (Broad Institute, UC San Diego) [15]. GSEA was performed to determine differentially expressed genes (DEGs) that were enriched in gene lists extracted from MSigDB v7.5.1 to determine enrichment in gene sets from the hallmark gene sets. Raw data files are available in the public data repository ‘ArrayExpress’ (Accession number: E-MTAB-13095).

### Public datasets

A gene-expression dataset of normal hematopoietic cells (GSE42519) was used to evaluate the expression pattern of *S100A9* at different stages of normal hematopoiesis [16]. In addition, a large dataset containing bulk AML samples and normal hematopoietic cell samples, (GSE13159) from BloodSpot (http://servers.binf.ku.dk/bloodspot/) was also used [17, 18]. For each microarray dataset, the probe set with the highest average expression was selected to represent *S100A9* expression level.

The Gene Expression Profiling Interactive Analysis (GEPIA) database (http://gepia2.cancer-pku.cn/), which was based on the UCSC Xena project, was used to analyze the mRNA expression status of *S100A9, S100A8, RAGE* and *TLR4* in AML [19, 20]. Briefly, mRNA expression data of 173 AML and 70 normal bone marrow samples from the Cancer Genome Atlas (TCGA) and Genotype-Tissue Expression (GTEx) were loaded into the server to analyze the differentially expressed genes between the two groups. The RNA-seq results were reported as the number of transcripts per million (TPM). The following filter indexes were set: expression mode was expression DIY, dataset was LAML, log-scale was log2 (TPM + 1), and *P*-value cut-off was 0.01. Then the mRNA expression parameters, including sample size and statistical box plot were displayed.

### Colony-Forming Unit (CFU) assay

To assess the clonogenic potential of AML cell lines (KG-1a, MOLM-13), AML cells were cultured in MethoCult media (H4100, M3231, STEMCELL Technologies, Inc., Canada) in the presence of dimethylsulfoxide (control) or tasquinimod. Cells were cultured in duplicate at a concentration of 1 × 10^4^ cells per well. Each well contained RPMI-1640 media consisting of Methylcellulose-Based Media, 10% FBS, 100 U/mL penicillin and 100 µg/mL streptomycin. Plates were incubated at 37 °C, 5% CO_2_ for 14 days. The colonies were photographed using the EVOS™ M7000 Imaging System (Thermo Fisher Scientific).

### Western blot

KG-1a and MOLM-13 cells were cultured at a density of 2.5 × 10^5^ cells/mL and were treated with tasquinimod for 48 h. For the combination experiment, AML cells were treated with tasquinimod for 24 h, followed by venetoclax treatment for additional 24 h. Cells were lysed in cell lysis buffer including protease (Roche) and phosphatase inhibitors (Sigma). Western blot analysis on these cell lysates was performed as previously described [21]. The following primary antibodies were used: p-mTOR (#5536), mTOR (#2983), p-P70S6K (#9204), P70S6K (#2708), p-S6K (#4868), S6K (#2217), p-4E-BP1 (#2855), 4E-BP1 (#9644), p-eIF2a (#9721), eIF2a (#9722), ATF4 (#11815), p21 (#2974), p27 (#3688), S100A9 (#72590), xCT/SLC7A11 (#12691), LAT1/SLC7A5 (#32683), BCL-2 (#2870), c-MYC (#5605) and horseradish peroxidase (HRP)-coupled anti-rabbit (#7074) and anti-mouse (#7076) secondary antibodies; all purchased from Cell Signaling Technology (Boston, MA). GRP78 (#sc-13968) was from Santa Cruz Biotechnology, anti-S100A8 (#MAB4570) from R&D, and anti-puromycin antibody, clone 12D10 (#618582) was from Sigma-Aldrich. The bands were visualized and captured using Pierce ECL Western Blot Substrate (Thermo Scientific) and LI-COR Odyssey Fc (Bad Homburg, Germany). Pixel densities were quantified using Image J.

### Mitochondrial oxygen flux and extracellular acidification analyses

Assessment of mitochondrial oxygen flux and extracellular acidification were measured using the Seahorse XFe96 Analyzer (Agilent Technologies, Santa Clara, CA, USA) using the Seahorse XFp Cell Mito Stress Test Kit (Agilent Technologies) according to manufacturer’s instructions. In brief, XFe96-well microplates (Agilent Technologies) were coated with Cell-Tak (Corning Inc., Corning, NY, USA) beforehand. The day of the assay, cells were washed and incubated in Seahorse RPMI assay medium supplemented with 2 mM glutamine and 1 mM pyruvate (all from Agilent Technologies). Cells were seeded at 6.0 × 10^4^ cells per well in the pre-coated XFe96-well microplates. Final inhibitor concentrations in each well were 1.5 μM oligomycin, 1 μM carbonyl cyanide-4-phenylhydrazone (FCCP), 0.5 μM antimycin A, and 0.5 μM rotenone. Oxygen consumption rate (OCR) and extracellular acidification rate (ECAR) were measured. After the experiment, cells were stained with 8 µM Hoechst solution (#33258, Sigma-Aldrich) for normalization. Data were automatically normalized by cell numbers quantified using a Cytation 1 Cell Imaging Reader (Agilent Technologies).

### Statistical analysis

Results (at least 3 replicates) were analyzed with GraphPad Prism 9 software (GraphPad Software Inc, La Jolla, CA, USA). A Mann–Whitney *U*-test and One-Way ANOVA was used to compare 2 groups or multiple groups, respectively. All data represent the mean ± standard deviation (SD). * *p* < 0.05; ** *p* < 0.01; *** *p* < 0.001, **** *p* < 0.0001, were considered statistically significant.

## Results

### S100A9 and its receptors are variably expressed across AML subtypes and AML cell lines

**Fig. 1:**
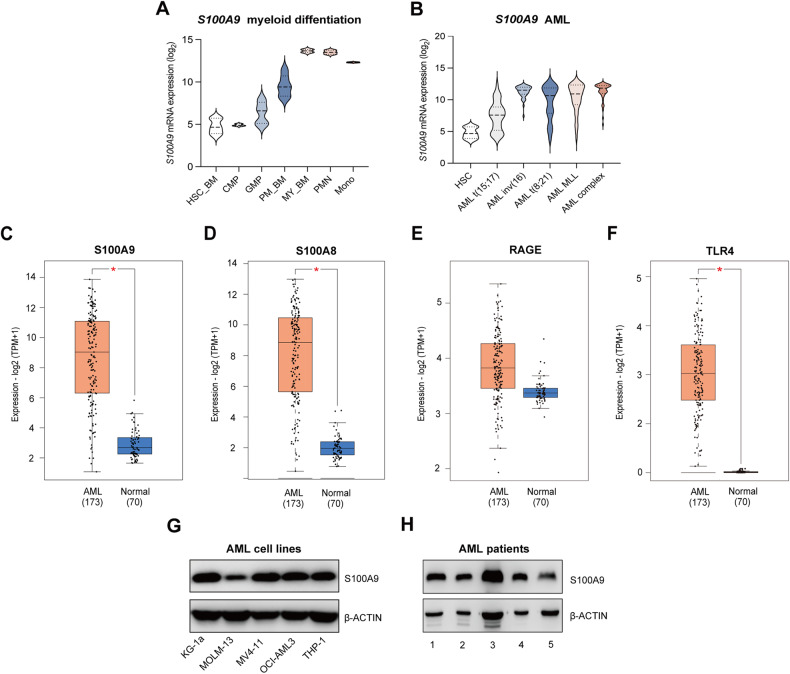
S100A9, S100A8, RAGE and TLR4 expression in AML patients and human AML cell lines. **A** Violin plots illustrating the expression pattern of *S100A9* during differentiation of the major hematopoietic lineages using published dataset (GSE42519). **B** Violin plot showing the expression pattern of *S100A9* in HSC and AML blasts using a merged data set (Bloodpool) from the BloodSpot website. **C**–**F** Box plot of the expression profile of *S100A9, S100A8, RAGE, TLR4* in AML (*n* = 173) and normal bone marrow samples (*n* = 70) (GEPIA). *p < 0.05, *t*-test was used to compare the difference in expression between tumor and normal tissues. **G**, **H** The protein level of S100A9 in different human AML cell lines and primary patient samples detected by western blot.

To evaluate *S100A9* mRNA expression levels during myeloid differentiation and in different AML subtypes, transcriptomic datasets were analyzed using the Bloodspot database [22]. *S100A9* was slightly expressed in normal hematopoietic stem cells (HSC) and was upregulated upon differentiation into monocytes and polymorphonuclear cells (Fig. 1A). *S100A9* was upregulated in AML blasts compared to HSC; however, a heterogeneous expression could be observed among the patients (Fig. 1B). While *S100A9*, its binding partner *S100A8* and its receptor *TLR4* were all significantly upregulated in AML patient compared to normal bone marrow sample, we observed no prognostic value for these genes in AML patients (Fig. 1C–F, Supplemental Fig. 1). Next, we determined the mRNA expression levels of *S100A8*, *S100A9* and its receptors *TLR4* and *RAGE* in human AML cell lines (Supplemental Fig. 2, Supplemental Table 1). While qRT-PCR showed some variability in expression levels among the tested cell lines, similar S100A9 protein levels could be detected using western blot, with the strongest expression for the KG-1a and the lowest expression for the MOLM-13 cells (Fig. 1G, Supplemental Fig. 2). Since KG-1a and MOLM-13 also belong to different subtypes, the M0/M1 (undifferentiated phenotype) and M5 (acute monocytic leukemia) subtype respectively according to the French, American, and British (FAB) classification system, these cell lines were selected for further experiments. Expression of S100A9 was also confirmed using primary BM or blood samples of 5 AML patients (Fig. 1H) (patients’ age, % of blasts and FAB subtype is included in Supplemental Table 2).

### S100A9-siRNA decreases AML cell viability and proliferation in vitro, associated with a dysregulated mTOR-ER stress signaling

**Fig. 2:**
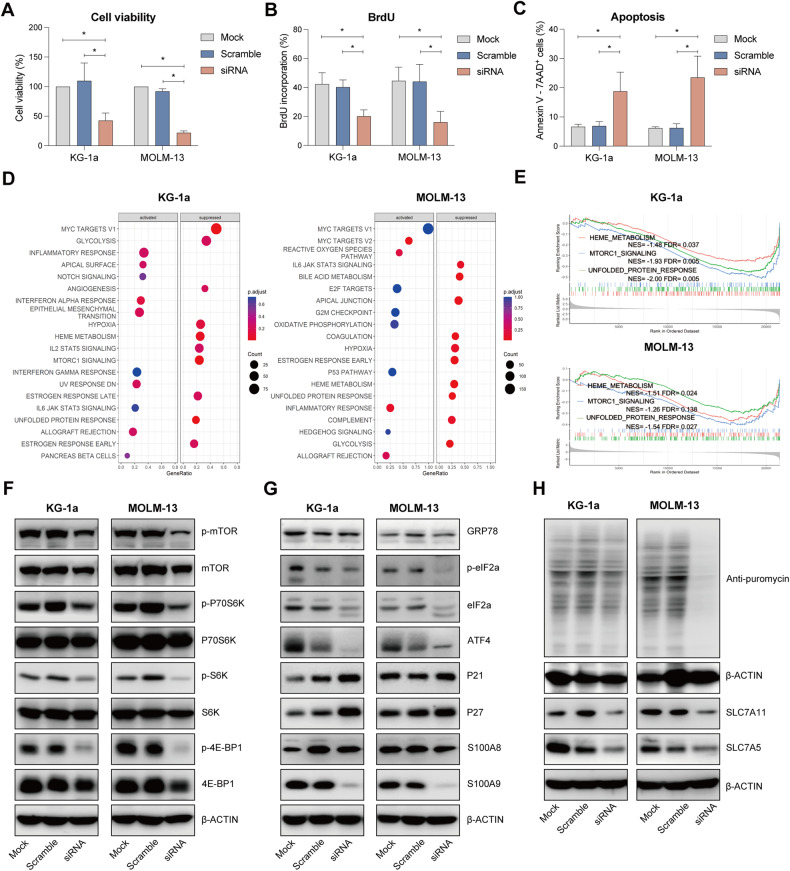
Effect of S100A9 silencing using S100A9-siRNA on cell viability, proliferation and apoptosis in human AML cell lines. KG-1a and MOLM-13 cells were exposed to 20 nM S100A9-siRNA and Lipofectamine 2000. A mock (only lipofectamine) and scramble condition (negative control) were included as controls. **A** Cell viability was detected by CellTiter-Glo at 72 h (*n* = 4). **B** Cell proliferation was investigated using BrdU staining at 72 h (*n* = 4). **C** Apoptosis was measured using an AnnexinV 7-AAD staining and flow cytometry at 72 h (*n* = 3). **D** RNA sequencing was performed on S100A9-siRNA treated KG-1a and MOLM13 cells at 72 h. The bubble plot shows the top 20 differentially regulated (activated/suppressed) pathways in the S100A9-siRNA group compared with mock (*n* = 3). **E** GSEA of the HEME_METABOLISM, MTORC1_SIGNALING and UNFOLD_PROTEIN_RESPONSE gene signature in KG-1a and MOLM-13 cells after treatment with 20 nM S100A9-siRNA for 72 h. GSEA of differentially expressed genes was determined by querying the MSigDB. False discovery rate (FDR) and normalized enrichment scores (NES) are indicated (*n* = 3). **F**–**H** KG-1a and MOLM-13 cells were cultured with 20 nM S100A9-siRNA for 72 h. Whole-cell lysates were subjected to Western blot analysis using anti-p-mTOR, mTOR, p-P70S6K, P70S6K, p-S6K, S6K, p-4E-BP1, 4EBP1, GRP78, p-eIF2a, eIF2a, ATF4, p21, p27, S100A9, puromycin, SLC7A11, SLC7A5 and anti-β-Actin antibodies (*n* ≥ 3). *p < 0.05, **p < 0.01, ***p < 0.001, ****p < 0.0001, One-way ANOVA, Error bars indicate SD.

To understand the functional role of S100A9 and its possible therapeutic implications in AML, we silenced the mRNA in KG-1a and MOLM-13 cells using S100A9-siRNA. Multiple siRNAs were tested and the most effective one was selected for further experiments (Supplemental Fig. 3A–C). After 72 h of silencing, we observed a significant reduction in cell viability and proliferation of KG-1a and MOLM-13 cells (Fig. 2A, B, gating strategy provided in Supplemental Fig. 4A), while the number of apoptotic cells was increased (Fig. 2C, gating strategy provided in Supplemental Fig. 4B). Moreover, cell cycle analysis revealed a significant decrease in the population of the siRNA-treated KG-1a and MOLM-13 cells in S-phase and an increase in the G1 phase (Supplemental Fig. 4C, D).

To obtain further mechanistic insights into the S100A9-mediated anti-tumoral effects in AML, we performed RNA sequencing on KG-1a and MOLM-13 cells, treated with S100A9-siRNA for 48 h. We found 299 up- and 255 downregulated genes in KG-1a, and 641 up- and 705 downregulated genes in MOLM-13 (data accessible in the public data repository ‘ArrayExpress’). Gene set enrichment analysis (GSEA) on the RNA-seq data (Fig. 2D, E) revealed a suppressed expression of genes associated with glycolysis, heme metabolism and the unfolded protein response in both cell lines. In KG-1a, MTORC1 signaling was also suppressed and could be linked to potential anti-tumor effects.

Using western blot analysis, we further validated the downstream effects of siRNA-mediated S100A9 gene silencing in both AML cell lines. S100A9-siRNA significantly downregulated S100A9 levels, however no reduction could be observed for its binding partner S100A8, suggesting the absence of a compensation mechanism. We observed a consistent downregulation of mTOR signaling (p-mTOR, p-p70-S6K, p-S6, p-4-E-BP1), ER stress signaling (p-eIF2, ATF4, GRP78), protein synthesis and a downregulation in amino acid transporters SLC7A5 and SLC7A11 (Fig. 2F–H, Supplemental Fig. 5). In addition, p21 and p27 were increased in both AML cell lines, indicative for a cell cycle arrest.

Altogether, these data illustrate the pro-tumoral effect of intracellular S100A9 in AML, regulating mTOR-ER stress signaling pathways which may contribute to tumor progression and drug resistance.

### S100A9 inhibitor tasquinimod reduces cell viability, proliferation and clonogenic potential of AML cells in vitro by targeting of mTOR-ER stress signaling

**Fig. 3:**
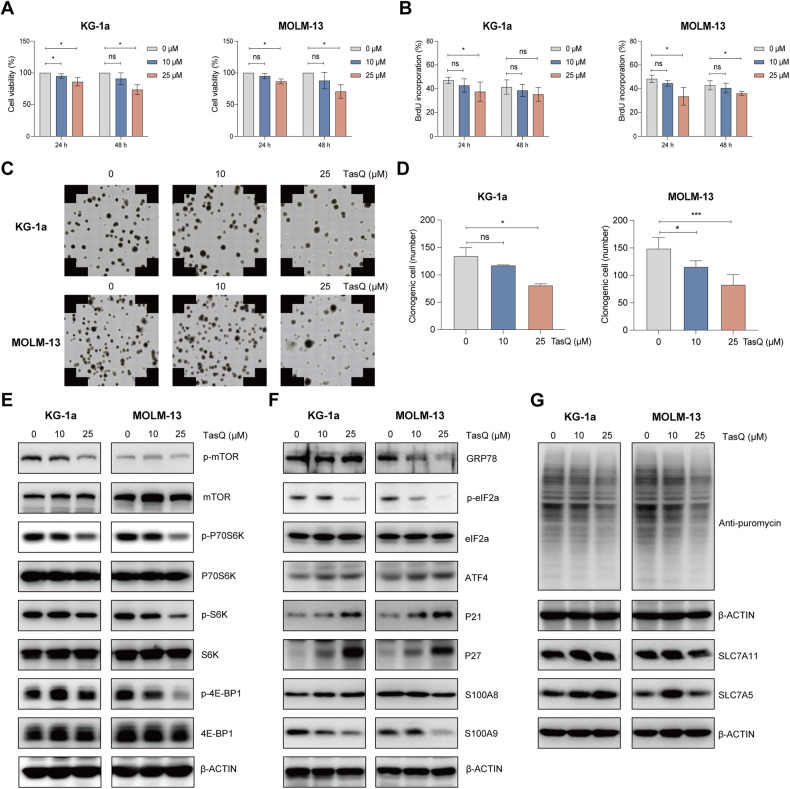
Tasquinimod inhibits AML cell proliferation and reduces colony formation by targeting of mTOR-ER stress signaling in vitro. **A** Cell viability of tasquinimod-treated KG-1a and MOLM-13 cell lines (10 and 25 µM) was analyzed using a CellTiter-Glo assay after 24 and 48 h (*n* = 4). **B** Cell proliferation of tasquinimod-treated AML cells (10, 25 μM) was investigated using BrdU staining after 24 and 48 h (*n* = 4). **C** Methylcellulose colony formation assays were performed for KG-1a and MOLM-13 cell lines treated with vehicle or tasquinimod (10, 25 μM) for 14 days. **D** Quantification of the colony numbers is shown (*n* = 4). **E**–**G** KG-1a and MOLM-13 cells were cultured with tasquinimod at indicated concentrations (10, 25 μM) for 48 h. Whole-cell lysates were subjected to Western blot analysis using anti-p-mTOR, mTOR, p-P70S6K, P70S6K, p-S6K, S6K, p-4E-BP1, 4E-BP1, GRP78, p-eIF2a, eIF2a, ATF4, p21, p27, S100A9, puromycin, SLC7A11, SLC7A5 and anti-β-Actin antibodies (*n* ≥ 3). *p < 0.05, **p < 0.01, ***p < 0.001, One-way ANOVA, Error bars indicate SD.

To unravel the potential of S100A9 as a therapeutic target in AML, we evaluated the anti-tumor effect of the clinically available S100A9 inhibitor tasquinimod. Tasquinimod is a quinoline-3-carboxamide that inhibits the interaction between S100A9 and its receptors [23, 24]. AML cell lines KG-1a and MOLM-13 were treated with increasing doses of tasquinimod for 24 h and 48 h. We observed a significant reduction in AML cell viability and proliferation, and an increase in apoptosis, at the highest concentration of 25 µM (Fig. 3A, B, Supplementary Fig. 6A–C). Using propidium iodide staining, we also observed a significant decrease in the number of cells in the S-phase for both cell lines (Supplemental Fig. 6D, E). Moreover, a 14-day treatment of AML cell lines with tasquinimod resulted in a significant reduction of the clonogenic capacity, as illustrated in Fig. 3C, D. Downstream analysis of signaling pathways revealed a reduced mTOR signaling in both AML cell lines (p-mTOR, p-P70S6K, p-4-EB-P1), reduced ER stress proteins (p-eIF2a) and decreased protein synthesis (Fig. 3E–G, Supplemental Fig. 7). Meanwhile, the p21 and p27 were increased in both AML cell lines, which is again indicative of a cell cycle arrest. However, the effects of tasquinimod on ATF4, GRP78 and amino acid transporters were less clear.

Taken together, tasquinimod affects AML cell survival and proliferation similar to gene silencing, by targeting mTOR-ER stress signaling, indicating the therapeutic potential of S100A9 as a novel target for the treatment of AML patients.

### Genetic and pharmacological inhibition of S100A9 differentially affect the cellular energy metabolism in vitro

**Fig. 4:**
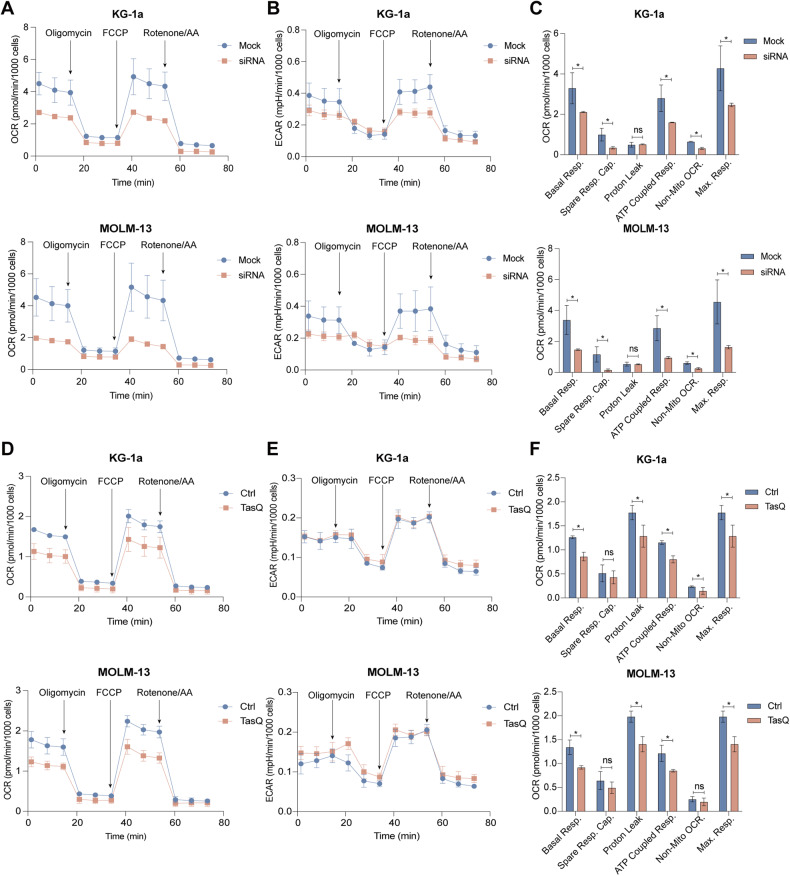
Seahorse analysis of cellular metabolic fluxes after siRNA-mediated knockdown or tasquinimod treatment in AML cells. Mitochondrial bioenergetics was analyzed using the Agilent XF Seahorse technology. KG-1a and MOLM-13 cells were treated with 20 nm S100A9-siRNA or tasquinimod (25 μM) for 48 h (*n* = 3). **A** OCR assessment of siRNA-transfected KG-1a (up) and MOLM-13 (down) cells. **B** ECAR assessment of siRNA-transfected KG-1a (up) and MOLM-13 (down) cells. **C** MitoStress test parameters of siRNA-transfected KG-1a and MOLM-13 cell lines. **D** OCR assessment of tasquinimod-treated KG-1a (up) and MOLM-13 (down) cells. **E** ECAR assessment of tasquinimod-treated KG-1a (up) and MOLM-13 (down) cells. **F** MitoStress test parameters of tasquinimod-treated KG-1a and MOLM-13 cell lines. *p < 0.05, **p < 0.01, ***p < 0.001, Mann–Whitney *U*-test, Error bars indicate SD.

Since mTOR signaling and the heme metabolism are closely associated with the mitochondrial metabolism and mitochondrial functions, we decided to explore the impact of S100A9 targeting on metabolic regulation of AML cells [25]. After S100A9-siRNA or tasquinimod treatment for 48 h, the Mito Stress test showed that treated KG-1a and MOLM-13 cells had a significantly lower basal and maximal respiration and ATP-coupled respiration compared to untreated AML cells; all indicative for reduced mitochondrial respiration and mitochondrial respiratory capacity (Fig. 4A–F). Interestingly, the non-mitochondrial OCR in siRNA treated cells was also reduced. The extracellular acidification rate (ECAR) was measured as an indicator of acidification, as a consequence of glycolysis and/or the tricarboxylic acid cycle (TCA) cycle, and showed a significant downregulation for siRNA-treated AML cell lines; while it remained unaffected using the S100A9 inhibitor tasquinimod (Fig. 4B, E). The AML cells “energy map” (based on OCR and ECAR) clearly demonstrated a shift in energy status from energetic to glycolytic (for tasquinimod) or quiescent (for S100A9-siRNA) after 48 h of therapy (Supplemental Fig. 8).

In conclusion, Seahorse data illustrate a difference in metabolic regulation of S100A9 in AML cells after genetic silencing (targeting intracellular S100A9) compared to pharmacological inhibition (targeting extracellular S100A9). While siRNA-mediated knockdown affected both OCR and ECAR, tasquinimod only affected the OCR in AML cells.

### SiRNA mediated targeting of S100A9 protein increases AML cell sensitivity to BCL-2 inhibitor venetoclax in vitro

**Fig. 5:**
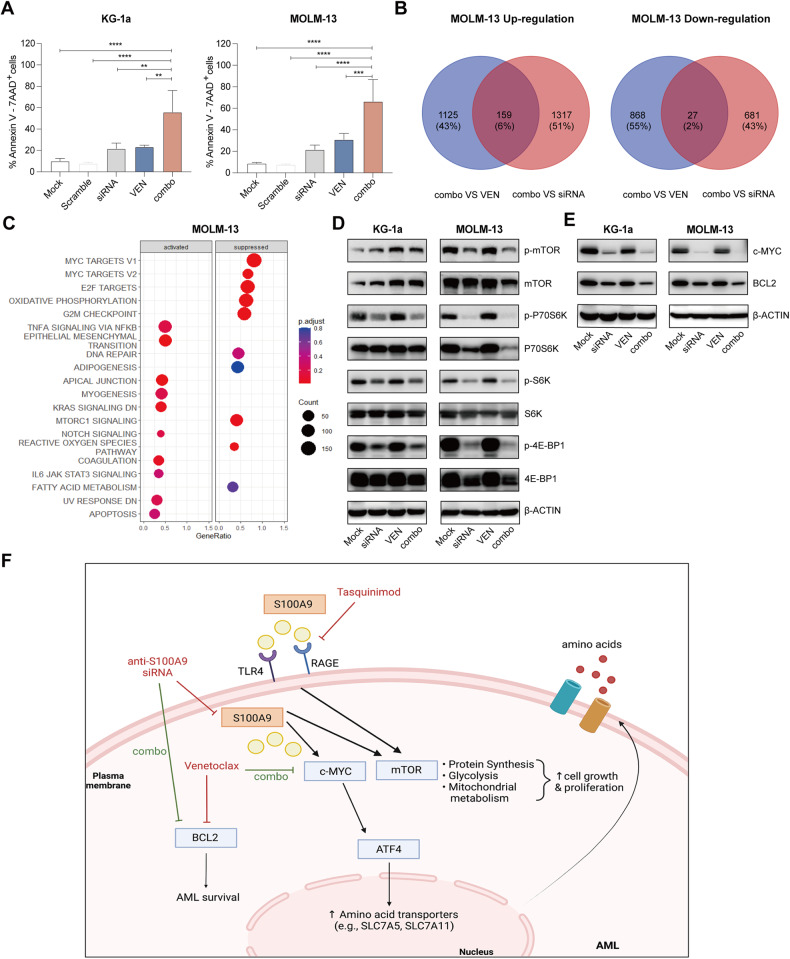
Combination of S100A9-siRNA with venetoclax has additive effects on AML cell apoptosis through downregulation of c-MYC and BCL2. S100A9-siRNA combined with venetoclax induced apoptosis of KG-1a and MOLM-13 cells. **A** KG-1a and MOLM-13 cells were pre-plated in RPMI medium with 10% fetal bovine serum in the presence or absence of siS100A9 (20 nM) for 48 h and add venetoclax (250 nM) co-culture for 24 h. Annexin-V/7-AAD based flow cytometric assay for determination of apoptotic cells. **B** Venn’s diagram of protein expression with different intensity between venetoclax vs combo and siRNA vs combo in MOLM-13. **C** The bubble plot shows the top 20 differentially regulated (activated/suppressed) pathways in combo group compared with siRNA (72 h) (*n* = 3). **D**, **E** KG-1a, MOLM-13 cells were cultured with tasquinimod at indicated concentrations (10, 25 μM) for 48 h. Whole-cell lysates were subjected to Western blot using anti-p-mTOR, mTOR, p-P70S6K, P70S6K, p-S6K, S6K, p-4E-BP1, 4EBP1, BCL2, c-MYC and anti-β-Actin antibodies (*n* ≥ 3). **F** Graphical abstract of siS100A9 and tasquinimod mediated effects, created with Biorender.com. *p < 0.05, **p < 0.01, ***p < 0.001, One-way ANOVA, Error bars indicate SD.

BCL-2 inhibitor venetoclax, combined with vidaza, emerged as a potent therapy for unfit AML patients [26]. However, approximately 30% of the patients do not respond to venetoclax therapy and many patients develop resistance while on treatment [27, 28]. A study by Guièze et al. demonstrated that metabolic modulators can cooperate with venetoclax to overcome resistance. Moreover, elevated expressions of S100A8 and S100A9 were previously described to correlate with resistance to venetoclax in AML [4, 29]. To better understand the link between S100A8/S100A9 levels and venetoclax, we first tested the effect of venetoclax therapy on S100A8 and S100A9 protein levels in KG-1a and MOLM-13 cells. We could not observe any significant effect of venetoclax therapy on both proteins after 24 h (Supplemental Fig. 9). Next, we decided to evaluate the anti-tumor effect of siRNA-mediated S100A9 knockdown (48 h) and venetoclax therapy (24 h) on KG-1a and MOLM-13 cells. After 48 h, we observed a significant increase in the number of apoptotic AML cells (>30%) compared to single agent therapy in both cell lines (Fig. 5A). RNA sequencing of MOLM-13 cells revealed 1284 upregulated and 895 downregulated genes comparing venetoclax to combination therapy (venetoclax + S100A9-siRNA), and 1476 upregulated and 708 downregulated genes comparing S100A9-siRNA to combination therapy (venetoclax + S100A9-siRNA) (Fig. 5B, Supplemental Fig. 10). GSEA on the overlapping genes revealed suppression of MYC targets, cell cycle regulators (e.g. E2F targets, G2M checkpoint) and mTORC1 signaling (Fig. 5C). Within the overlapping gene list, we also found a downregulation of BCL-2 using the combination therapy compared to single agent therapy. Using western blot analysis, we validated the effect on downstream targets and could observe a significant reduction in c-MYC and BCL-2 expression using S100A9-siRNA combined with venetoclax compared to single agent treatment of both AML cell lines (Fig. 5D, E, Supplemental Fig. 11). The observed effects of S100A9-siRNA combined with venetoclax therapy on downstream targets in AML are graphically summarized in Fig. 5F.

### SiRNA and tasquinimod-mediated targeting of S100A9 has anti-leukemic activity in venetoclax-resistant AML cell lines

**Fig. 6:**
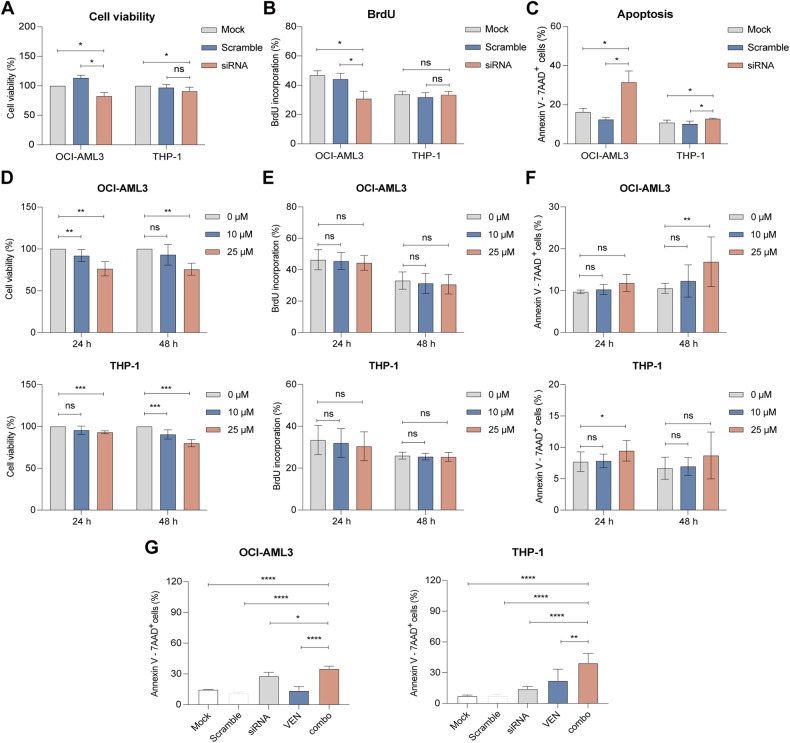
Anti-leukemic effect of S100A9-siRNA and tasquinimod on cell viability, proliferation and apoptosis in venetoclax-resistant AML cell lines. Venetoclax-insensitive OCI-AML3 and THP-1 cells were treated with 20 nM (OCI-AML3) or 60 nM (THP-1) S100A9-siRNA and Lipofectamine 2000. A mock (only lipofectamine) and scramble condition (negative control) were included as controls. **A** Cell viability was measured by CellTiter-Glo at 72 h (*n* = 4). **B** Cell proliferation was investigated using BrdU staining at 72 h (*n* = 4). **C** Apoptosis was measured using an AnnexinV 7-AAD staining and flow cytometry at 72 h (*n* = 3). **D** Cell viability of tasquinimod-treated OCI-AML3 and THP-1 cell lines (10 and 25 µM) was analyzed using a CellTiter-Glo assay after 24 and 48 h (*n* = 4). **E** Cell proliferation of tasquinimod-treated AML cells (10, 25 μM) was investigated using BrdU staining after 24 and 48 h (*n* = 4). **F** Apoptosis was measured using an AnnexinV 7-AAD staining and flow cytometry after 24 and 48 h (*n* = 4). **G** OCI-AML3 and THP-1 cells were pre-plated in RPMI medium with 10% fetal bovine serum in the presence or absence of S100A9-siRNA (20 nM) for 48 h and afterwards venetoclax (250 nM) was added for an additional 24 h. The Annexin-V/7-AAD based flow cytometric was used to determine the % of apoptotic cells. (*p < 0.05, **p < 0.01, ***p < 0.001, ****p < 0.0001, One-way ANOVA, Error bars indicate SD).

To evaluate whether S100A9 targeting had a therapeutic effect on AML resistant cell lines, we tested the anti-leukemic effect of S100A9-siRNA and tasquinimod on venetoclax-insensitive cell lines THP-1 and OCI-AML3. The transfection efficiency and the downregulation of S100A9 protein levels was validated for both cell lines (Supplemental Fig. 12A–D). We observed a significant reduction in cell viability and an increase in apoptosis using S100A9-siRNA (Fig. 6A–C, Supplemental Fig. 13) and tasquinimod (Fig. 6D–F, Supplemental Fig. 14). However, the effects were less pronounced compared to the venetoclax-sensitive cell lines MOLM-13 and KG-1a, and no clear anti-proliferative effect could be observed (Fig. 6B and E). Interestingly, we could observe a significant increase in apoptosis using the combination of siRNA and venetoclax in both AML cell lines (Fig. 6G).

### Tasquinimod increases venetoclax sensitivity in AML cell lines and primary patient samples

**Fig. 7:**
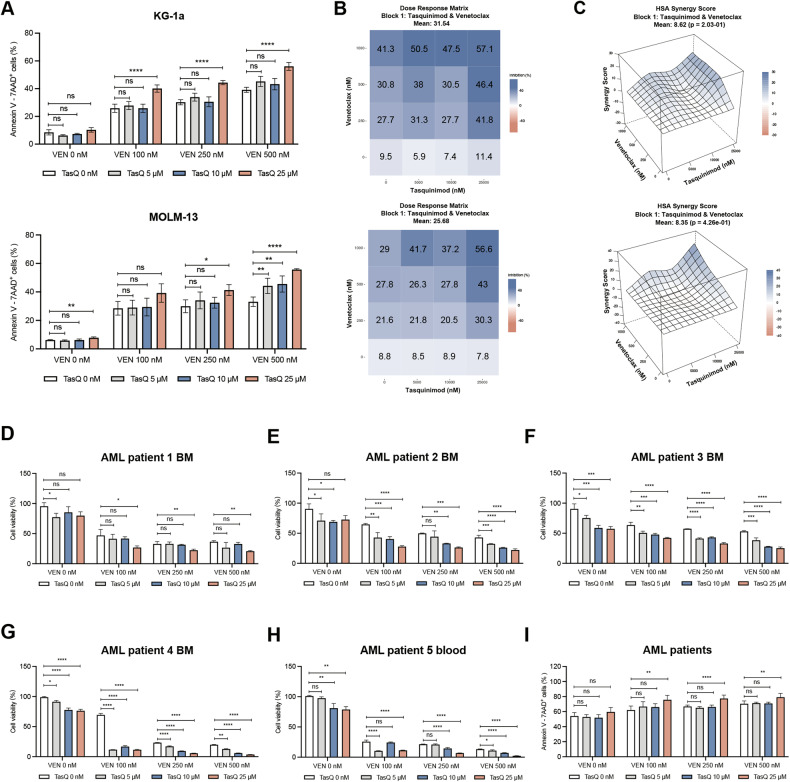
The effect in combination of TasQ and venetoclax in vitro and in primary AML patient samples. **A** KG-1a and MOLM-13 cell lines were pre-treated with tasquinimod (5, 10, 25 μM) for 24 h, combined with venetoclax (100, 250, 500 nM) for 24 h. Cell apoptosis was determined using an Annexin V/7-AAD staining and flow cytometry. **B**, **C** The effect of the drug combination (synergism, additive effects or antagonism) was calculated and visualized using SynergyFinder plus software and the HSA (Highest Single Agent) reference model. Blue regions - synergism; white - additive effect; pink - antagonism (*n* = 4). **D**—**H** AML patients BMMCs or PBMCs were pre-treated with TasQ (5, 10, 25 μM) for 24 h, combined with venetoclax (10, 20, 50 nM) for 24 h. Cell viability was determined by CellTiter-Glo (*n* = 3/sample). **I** Apoptosis was measured using flow cytometry and summarized in one graph for all tested samples (*n* = 5). *p < 0.05, **p < 0.01, ***p < 0.001, One-way ANOVA, Error bars indicate SD.

To demonstrate the therapeutic potential of S100A9 inhibitor tasquinimod in combination with venetoclax as a novel therapeutic approach for unfit/frail AML patients, we tested the combination on KG-1a and MOLM-13 cell lines, and 5 primary patient samples with variable tumor loads (Supplemental Table 2). We could observe a significant increase in KG-1a and MOLM-13 cell apoptosis (and reduced cell viability), and synergistic effects could be determined for multiple drug concentrations using the highest single-agent (HSA) method (Fig. 7A, B, Supplemental Fig. 15A–C). Combination therapy in primary AML patient samples could significantly reduce AML cell viability compared to single agent treatment, even at low doses of tasquinimod and venetoclax (Fig. 7D–H). This reduction in cell viability was also associated with a significant induction of apoptosis (Fig. 7I). The calculated synergistic effects in patient samples were even more pronounced than those observed in human AML cell lines (Supplemental Fig. 16A–F). As a control condition, we also tested the combination of tasquinimod and venetoclax on healthy donor derived PBMCs and healthy stromal HS-5 cells. Although some minor effects were observed on cell viability, we could not observe any increase in apoptosis of HS-5 and PBMCs (Supplemental Fig. 17A, B).

### Conclusion and discussion

Adult AML is characterized by high rates of relapse, and unfit patients are typically confronted with limited treatment options [30]. This study identified S100A9 as an interesting molecular target for the treatment of AML patients. Targeting the S100A9 protein, either by siRNA or using a small molecule inhibitor, resulted in reduced AML cell viability and proliferation. Moreover, using the Seahorse analyzer, we observed a dysregulated energy metabolism in S100A9-siRNA and tasquinimod-treated AML cells. Interestingly, besides its clear single agent anti-tumor effect, our data also demonstrated the therapeutic potential of S100A9-targeting therapies in combination with standard-of-care agent venetoclax.

Prior research in both solid tumors and hematological cancers already demonstrated an important role of S100A9 in cancer progression and drug resistance (e.g. chemotherapy, radiotherapy and immunotherapy resistance) [31]. Overexpression of the S100A9 protein has been linked to poor prognosis in non-small cell lung cancer, renal cell carcinoma, hepatocellular carcinoma and breast cancer [32–35]. Although we could not observe a prognostic value of S100A9 in AML patients, we found a significant increase in S100A9 expression in AML samples compared to normal tissue. Laouedj et al. previously assessed the effect of recombinant S100A8 and S100A9 in myeloid differentiation and AML cell proliferation. Interestingly, they found that S100A9 could promote AML cell differentiation (through TLR4) and in vivo administration of recombinant S100A9 protein could prolong the survival of leukemic mice [12]. In contrast to these data, we found that siRNA- and tasquinimod-mediated targeting of S100A9 could significantly reduce AML cell survival and proliferation. Further studies are required to fully understand these opposing roles of S100A9 in AML pathogenesis and tumor progression.

In our study, we also provided additional mechanistic insights on how targeting of intracellular and extracellular S100A9 protein could modulate downstream signaling pathways in AML. Previous studies demonstrated an effect of S100A9 on PI3K, AKT, NF-kB and mTOR signaling in multiple normal and malignant cell types [36–38]. Using RNA sequencing and western blot analysis, we could confirm a reduction in mTOR and ER stress signaling pathways in siRNA- and tasquinimod-treated AML cell lines. Moreover, targeting S100A9 resulted in a clear metabolic dysregulation of AML cells by reducing cellular respiration. Although metabolic modulators are considered as a promising treatment option for AML patients and are currently evaluated in clinical trials, they are often associated with a high toxicity profile [39–41]. The S100A9 inhibitor tasquinimod already demonstrated both efficacy and a favorable toxicity profile in castrate-resistant prostate cancer (NCT01732549, NCT01234311) and might offer an alternative and safer approach to disturb the cancer cell metabolism.

Although venetoclax, combined with vidaza, is an effective therapy for elderly AML patients, both intrinsic and acquired resistance are a major clinical challenge in AML [42]. A study by Karjalainen and colleagues found that high expression of *S100A8* and *S100A9* in AML is linked to venetoclax resistance. However, knockdown of *S100A8* and *S100A9* in the resistant cell lines OCI-AML3 and SHI-1 had no effect on venetoclax-induced apoptosis [43]. In contrast to this study, we observed an increased sensitivity towards venetoclax using S100A9-siRNA in venetoclax-sensitive (KG-1a and MOLM-13) and venetoclax-insensitive (OCI-AML3 and THP-1) AML cell lines, and in primary patient samples. Collectively, these data indicate that S100A9 inhibition might be a therapeutic option to increase venetoclax sensitivity, but further studies on venetoclax sensitive and insensitive patient samples will be needed to support this hypothesis.

Altogether, this study identified S100A9 as a promising target for the treatment of AML. Our study fosters the evaluation of the S100A9 inhibitor tasquinimod, already in clinical trial for the treatment of relapsed/refractory multiple myeloma patients, in AML, as a single agent or in combination with venetoclax.

## Availability of the data and materials

The raw data generated in this study are available upon request, from the corresponding author. Raw data files of the RNA-sequencing are available in the public data repository ‘ArrayExpress’ (Accession number: E-MTAB-13095).

### Supplementary information

Supplementary Information
