# Supplementary material for: Targeting S100A9 protein affects mTOR-ER stress signaling and increases venetoclax sensitivity in Acute Myeloid Leukemia
Source: Blood Cancer J. 2023 Dec 18;13(1):188. doi: 10.1038/s41408-023-00962-z (PMC10728073; doi:10.1038/s41408-023-00962-z)
Supplement: Supplementary file 1 — Supplementary Information [file 41408_2023_962_MOESM1_ESM.docx]

**Targeting intracellular and extracellular S100A9 protein affects mTOR-ER stress signaling pathways and increases venetoclax sensitivity in Acute Myeloid Leukemia**

Rong Fan^1,2^, Hatice Satilmis^1,2^, Niels Vandewalle^1,2^, Emma Verheye^1,2,3^, Elke De Bruyne^1,2^, Eline Menu^1,2^, Nathan De Beule^4^, Ann De Becker^4^, Gamze Ates^5^, Ann Massie^5^, Tessa Kerre^6^, Marie Törngren^7^, Helena Eriksson^7^, Karin Vanderkerken^1,2^, Karine Breckpot^2,8^, Ken Maes^1,9^, Kim De Veirman^1,2,4^

**Supplementary material and methods**

**Cell culture**

HS-5 and healthy PBMC were maintained in RPMI-1640 medium (Gibco; Thermo Fisher Scientific, Inc., Waltham, MA, USA).

**RNA isolation, cDNA synthesis and Real-time PCR**

The total RNA was extracted using RNeasy mini kit (QIAGEN, Hilden, Germany) and converted to cDNA by the Verso cDNA Synthesis Kit (Thermo Fisher Scientific, Waltham, MA, USA). The expression level of mRNA was quantified by real-time PCR using the QuantStudio 12 K Flex Real-Time PCR System (Thermo Fisher Scientific). ABL was included as an internal control. Relative mRNA expression normalized to ABL was carried out using the 2^-ΔΔCt^ method. Gene specific primers were purchased at Integrated DNA Technologies (Leuven, Belgium). Primer sequences are listed in Supplementary Table 1.

**Survival analysis**

Gene Expression Profiling Interactive Analysis (GEPIA) (http://gepia.cancer-pku.cn/index.html) is an analysis tool that contains RNA sequencing expression data of 9,736 tumors and 8,587 normal samples from TCGA and the GTEx projects. In this study, we evaluated the prognostic value of *S100A9, S100A8, RAGE, TLR4* expression in AML patients and healthy donors. The P value cutoff was set at 0.05. Prognostic analysis was performed using a Kaplan-Meier curve.

**Supplementary Table**

| ***Gene*** |  | **Primer sequence 5′-3′** |
| --- | --- | --- |
| ***ABL*** | Forward  Reverse | CCG TGG GTG CCA CTA TAT TT  GGG CAC AGT GGT GAA CTA TT |
| ***S100A9*** | Forward  Reverse | ACA GCA AAG CAG ACA GAA CTA  GAA AGG AAC TGC TGG GAT ACA |
| ***S100A8*** | Forward  Reverse | CCC AGT GAA TAC TGA TGA GAC C  GGA GGG ATA GTT AGC TGG ATT G |
| ***RAGE*** | Forward  Reverse | GGA ATC AAG GGC ACA GAG TTA  TTC CAT CTG CTC CAC AAT CC |
| ***TLR4*** | Forward  Reverse | GGA ATC TTG GAG CGA GTT GT  CCT CTT GTC TTT GAC CCA GTA G |

**Supplementary Table 1. Primer sequences used for real-time PCR.**

| **Patient No.** | **Gender** | **Age (year)** | **Diagnosis** | **Origin** | **Blasts (% flow)** | **Blast (% morfo)** |
| --- | --- | --- | --- | --- | --- | --- |
| ***1*** | M | 76 | AML-M1 | BM | 44 % | 37 % |
| ***2*** | M | 72 | AML-M2 | BM | 40 % | 54 % |
| ***3*** | F | 66 | AML- inv (16), KITmut | BM | 49.5 % | 64 % |
| ***4*** | M | 76 | AML-M0 | BM | 95 % | 92 % |
| ***5*** | M | 76 | AML-M0 | Blood | 73 % | 69 % |

**Supplementary Table 2. AML patient’s characteristics. Supplementary Figures**


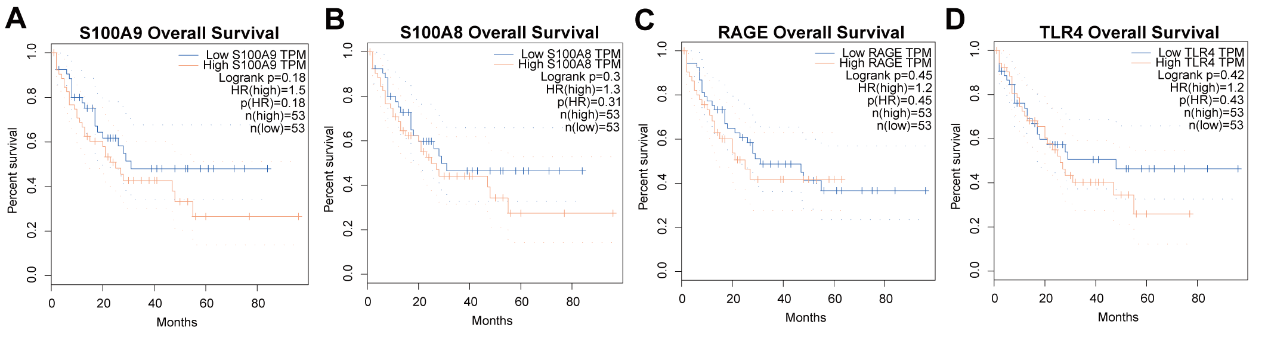


**Supplemental Figure 1. The prognostic value of *S100A9*, *S100A8*, *RAGE* and *TLR4* in the TCGA-LAML cohort (GEPIA; n = 106).** (**A**) The overall survival (OS) curves of AML patients with high and low S100A9 expression. (**B**) The OS curves of AML patients with high and low S100A8 expression. (**C**) The OS curves of AML patients with high and low RAGE expression. (**D**) The OS curves of AML patients with high and low TLR4 expression. OS analysis was performed using the Kaplan-Meier method, displayed with hazard ratios (HRs) and compared using log-rank tests. *p* < 0.05 indicated statistically significant differences.


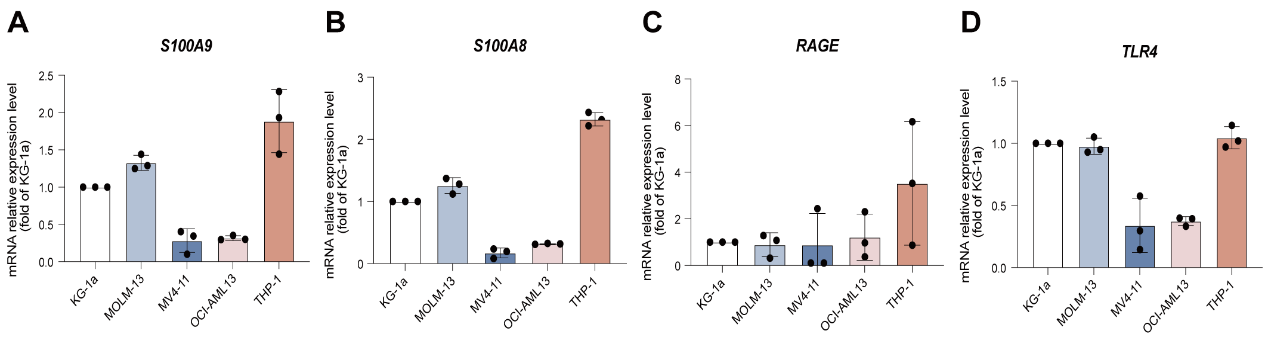


**Supplemental Figure 2. *S100A9*, *S100A8*, *RAGE* and *TLR4* gene expression in human AML cell lines.** Expression of S100A9, S100A8, RAGE and TLR4 was assessed using real-time PCR (n = 3).

**
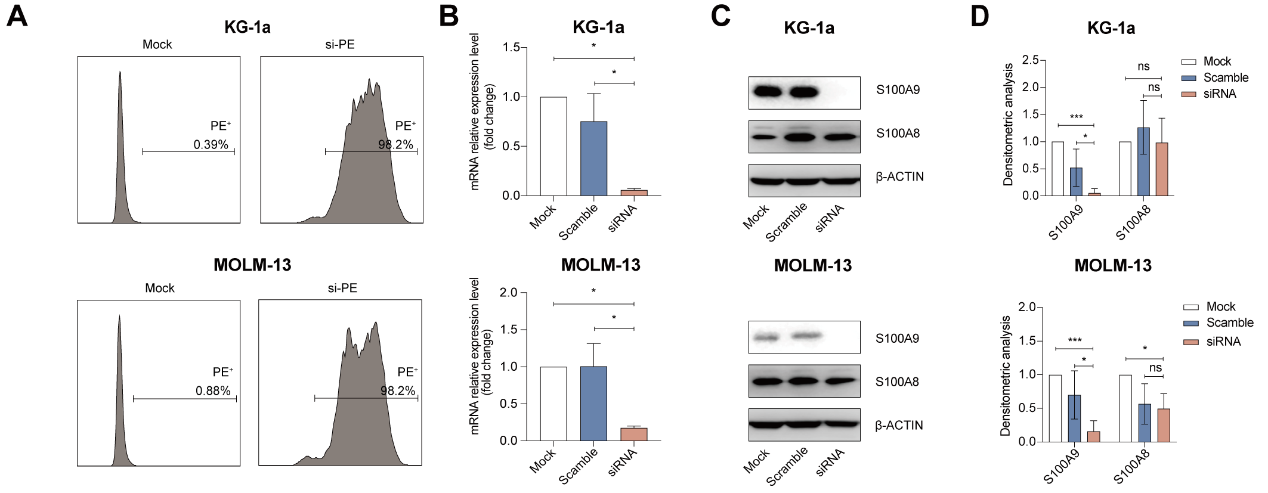
**

**Supplemental Figure 3. Transfection efficiency and S100A9 silencing using S100A9-siRNA in KG-1a and MOLM-13 AML cell lines.** (**A**) KG-1a and MOLM-13 cells were incubated with 20 nM PE- labeled siRNA and Lipofectamine 2000. After 24 hours, cells were analyzed by flow cytometry to assess the transfection efficiency. (n = 1) (**B-C**) KG-1a and MOLM-13 cells were transfected with multiple S100A9-siRNAs using 20 nM siRNA and Lipofectamine 2000. A mock (only lipofectamine) and scramble condition (negative control) were included as controls. S100A9 mRNA (48 hours after transfection) and protein levels (72 hours after transfection) were measured using real-time PCR and western blot respectively. ABL (for real-time PCR) and β-ACTIN (for western blot) were included as internal controls. (**D**) Grayscale analyses of western blots using ImageJ software. (n = 3) (**p* < 0.05, ****p* < 0.001, One-way ANOVA, Error bars indicate SD).


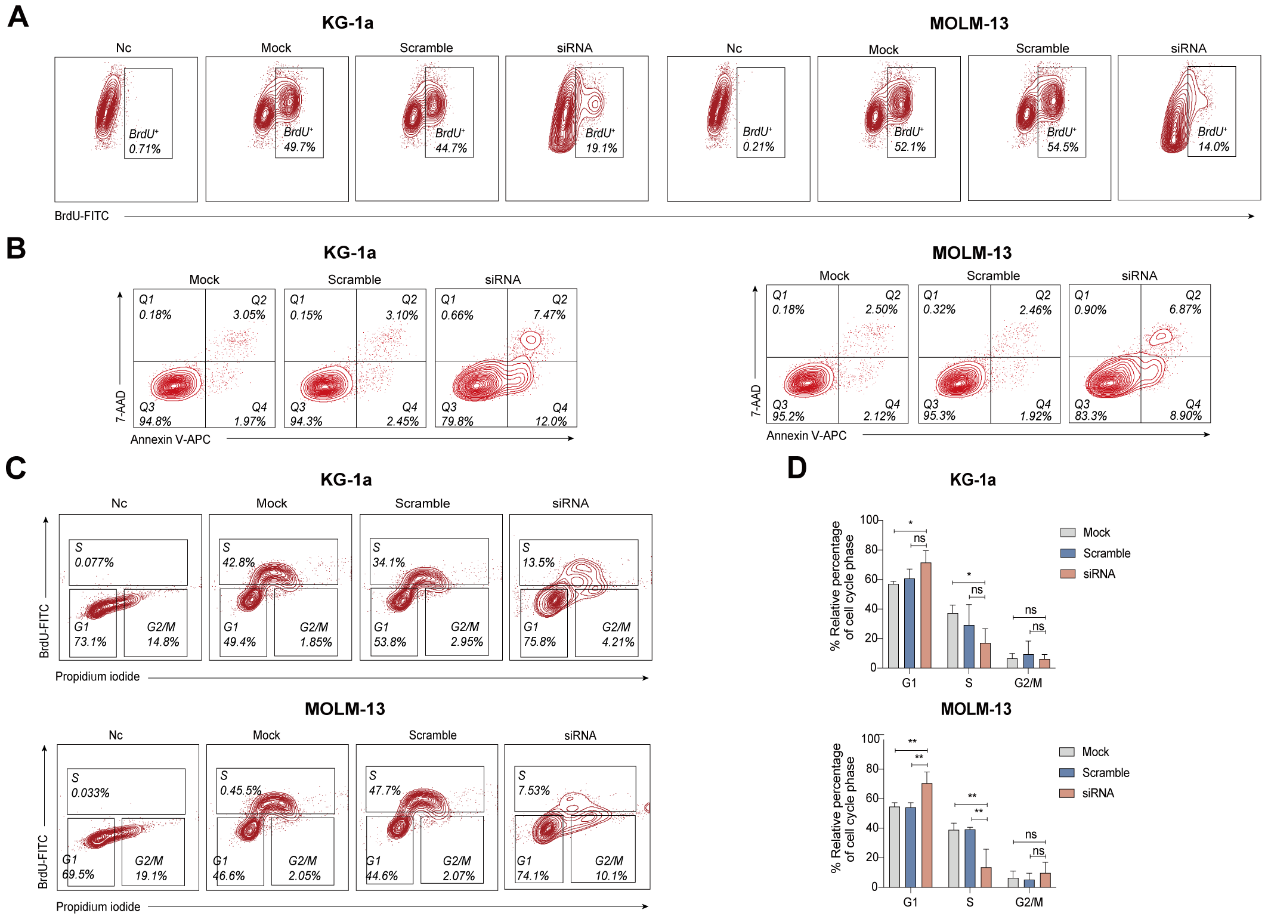


**Supplemental Figure 4. Effect of S100A9-siRNA on cell viability, proliferation and apoptosis of AML cell lines.** KG-1a and MOLM-13 cells were exposed to 20 nM siRNA S100A9 and Lipofectamine 2000 for 48 hours (n = 4). A mock (only lipofectamine) and scramble condition (negative control) were included as controls. (**A**) Cell proliferation was analyzed using a BrdU staining and flow cytometry. Representative figures and the gating strategy are shown. (**B)** Apoptosis was detected by flow cytometry using an Annexin V/7-AAD staining and representative figures (including gating strategy) are shown. (**C, D**) Cells cycle analysis was assessed using a BrdU/Propidium iodide staining. Representative figures and the gating strategy are shown. (**p* < 0.05, ***p* < 0.01, One-way ANOVA, Error bars indicate SD).

**
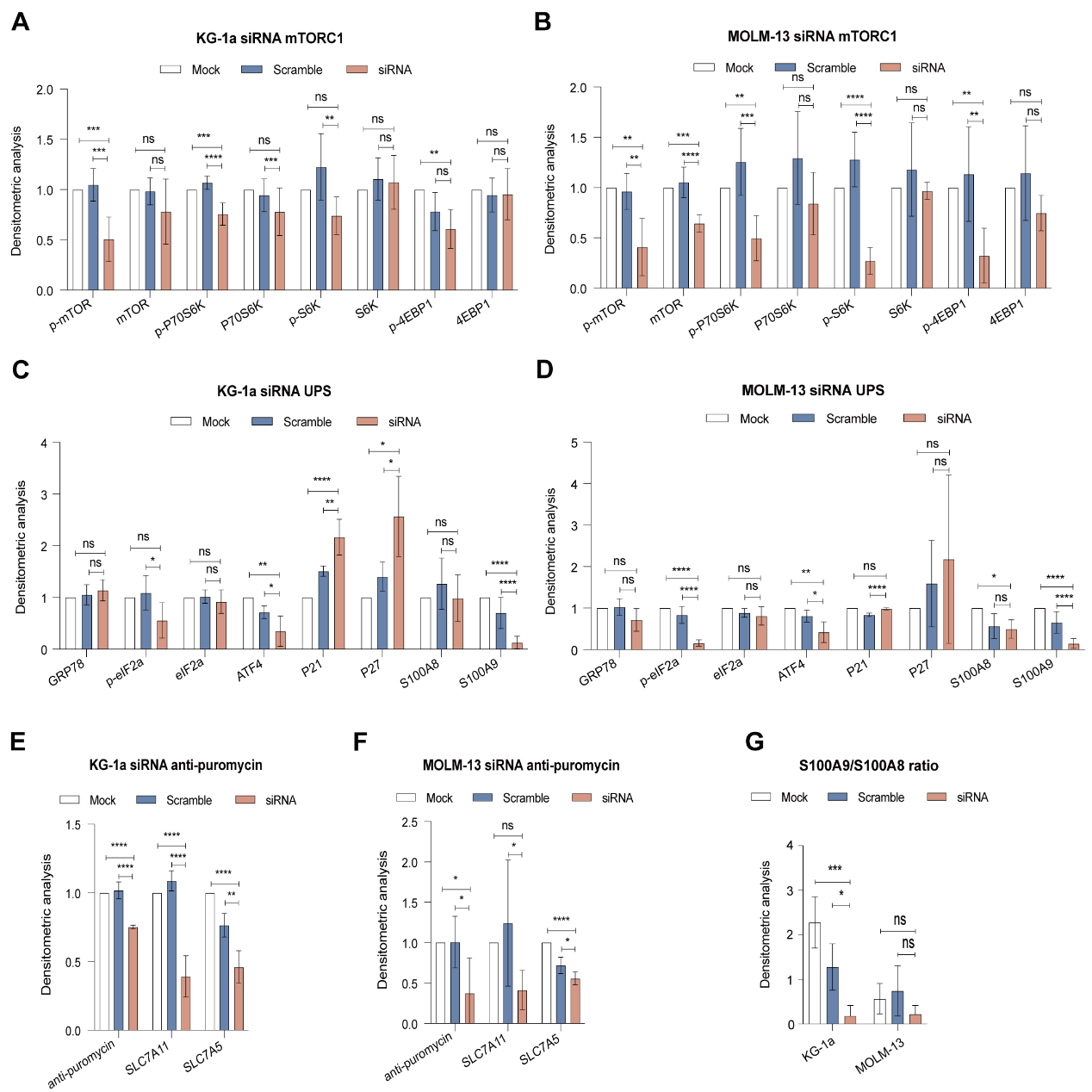
**

**Supplemental Figure 5. Grayscale analyses of western blots in Figure 2.** KG-1a and MOLM-13 cells were treated with 20 nM S100A9-siRNA for 48 hours (n ≥ 3). Western blot signals were analyzed using ImageJ software. (**p* < 0.05, ***p* < 0.01, ****p* < 0.001, *****p* < 0.0001, One-way ANOVA, Error bars indicate SD).

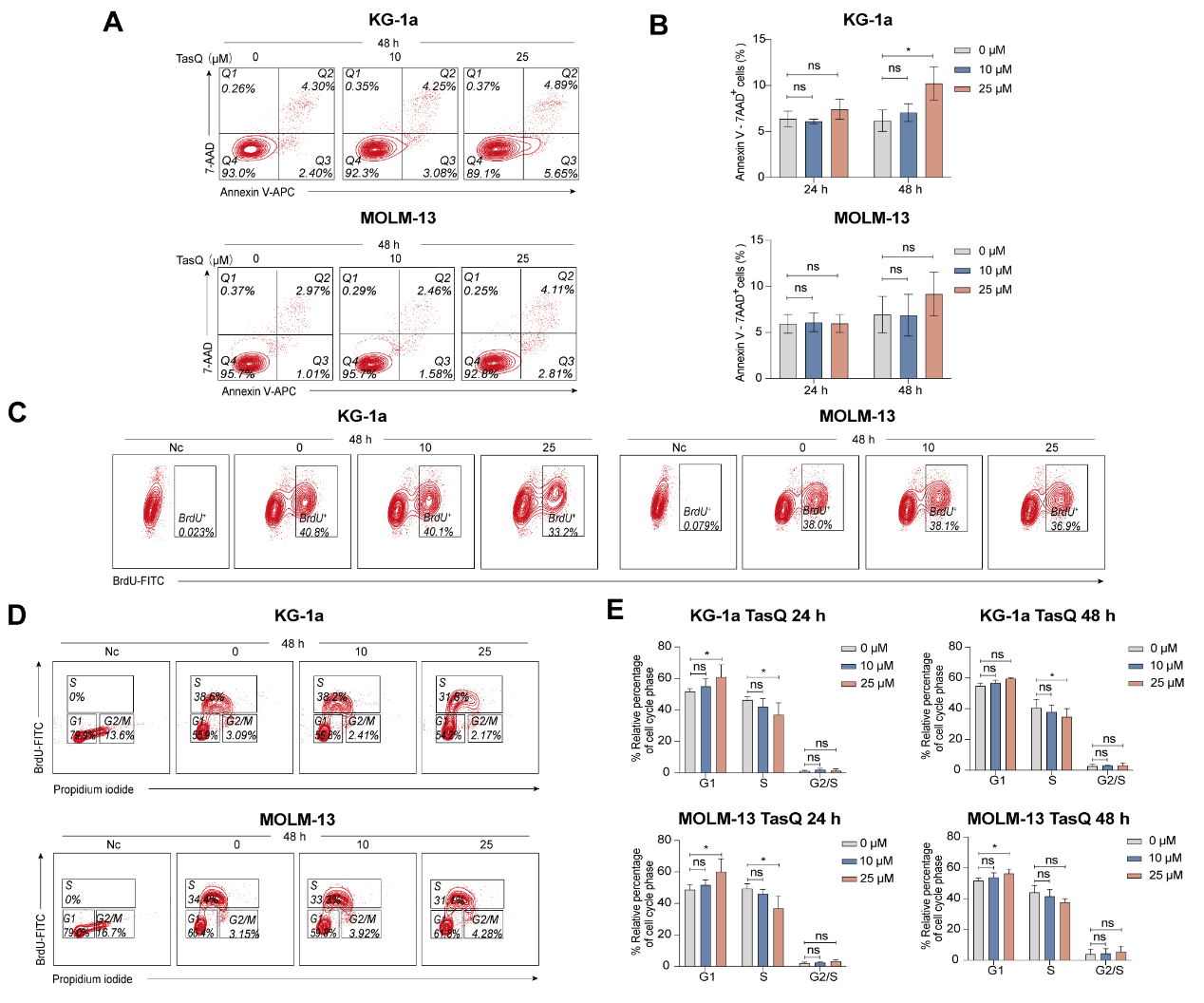


**Supplemental Figure 6. Effect of tasquinimod on cell apoptosis and proliferation of human AML cell lines.** KG-1a and MOLM-13 cells were exposed to different concentrations (0, 10, 25 μM) of tasquinimod for 24 and 48 hours (n = 4). (**A, B**) Cell apoptosis was detected by flow cytometry using an AnnexinV/7-AAD staining and representative figures (including gating strategy) are shown. (**C**) Cell proliferation was analyzed using BrdU staining and flow cytometry. Representative figures and the gating strategy are shown. (**D, E**) Cells cycle analysis was assessed using BrdU/ Propidium iodide staining. Representative figures and the gating strategy are shown. (**p* < 0.05, One-way ANOVA, Error bars indicate SD).

**
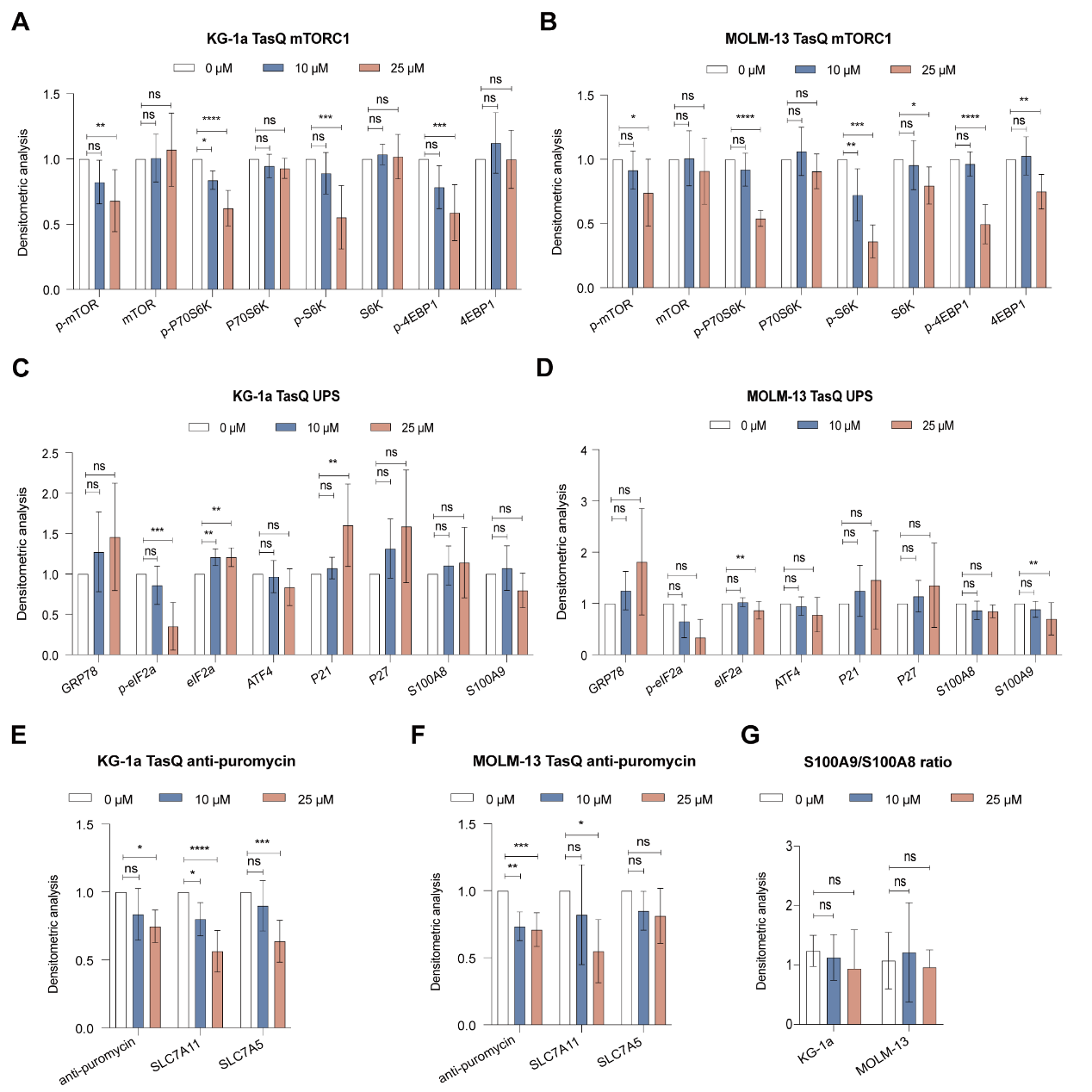
**

**Supplemental Figure 7. Grayscale analyses of western blots in Figure 3.** Human AML cell lines KG-1a and MOLM-13 were treated with different concentrations of tasquinimod (10, 25 μM) for 48 hours (n ≥ 3). Western blot signals were analyzed using ImageJ software. (**p* < 0.05, ***p* < 0.01, ****p* < 0.001, *****p* < 0.0001, One-way ANOVA, Error bars indicate SD).

**
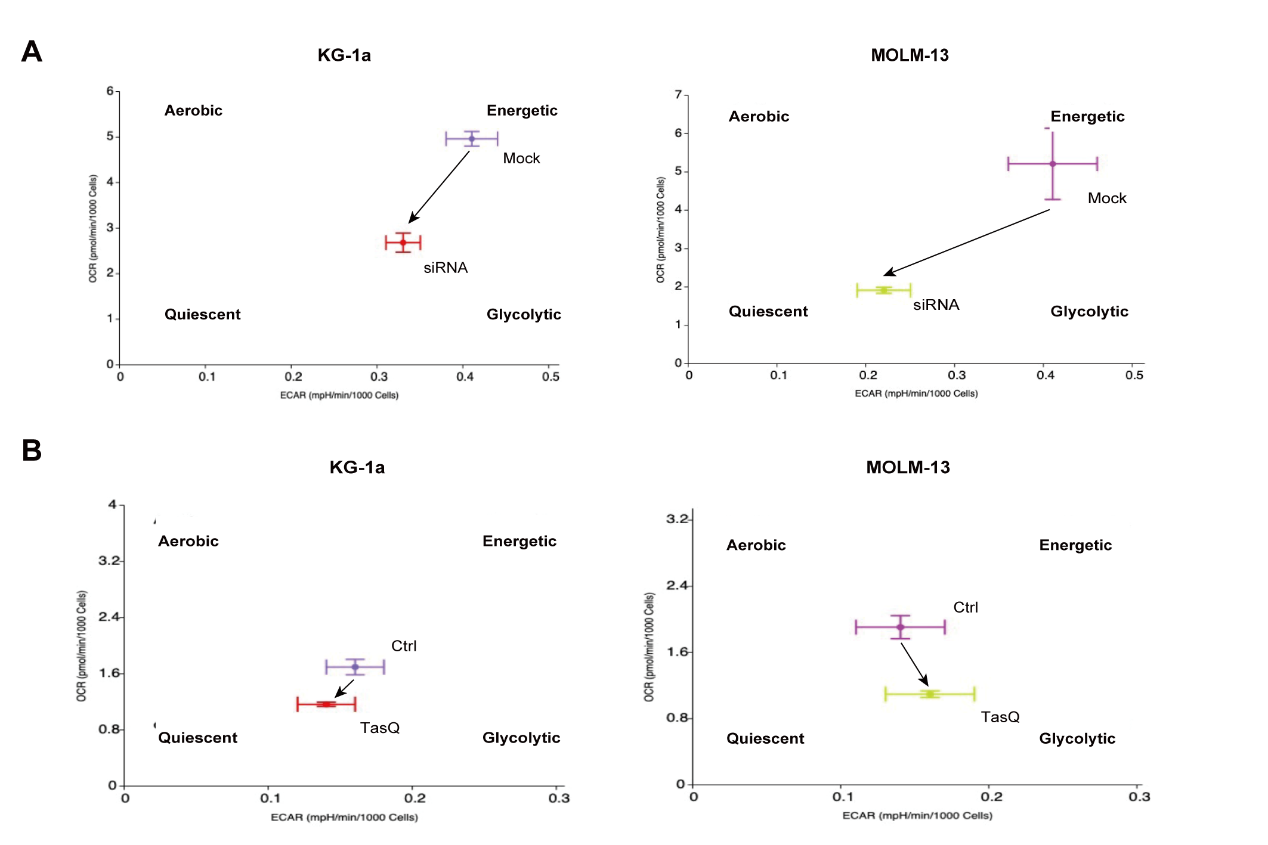
**


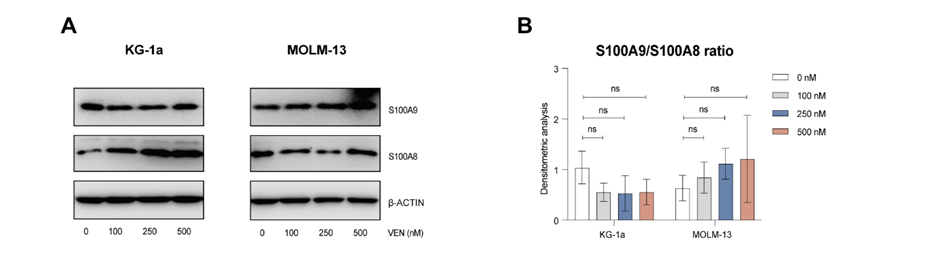
**Supplemental Figure 8. Energy maps of S100A9-siRNA and tasquinimod-treated AML cell lines.** (**A**) Energy maps were calculated after treatment of KG-1a and MOLM-13 cells with S100A9-siRNA for 48 hours. (**B**) Energy maps were calculated after treatment of KG-1a and MOLM-13 cells with 25 μM tasquinimod for 48 hours. (n = 3, Error bars indicate SD).

**Supplemental Figure 9.** **The effect of venetoclax on S100A8 and S100A9 levels in KG-1a and MOLM-13 cells.** **(A)** Cells were treated at indicated concentrations for 24h and protein levels of S100A8, S100A9 and B-actin were determined using western blot analysis. **(B)** Quantitative analysis of the western blots in KG-1a and MOLM-13 cells (n = 3). (ns = not significant, One-way ANOVA, Error bars indicate SD).


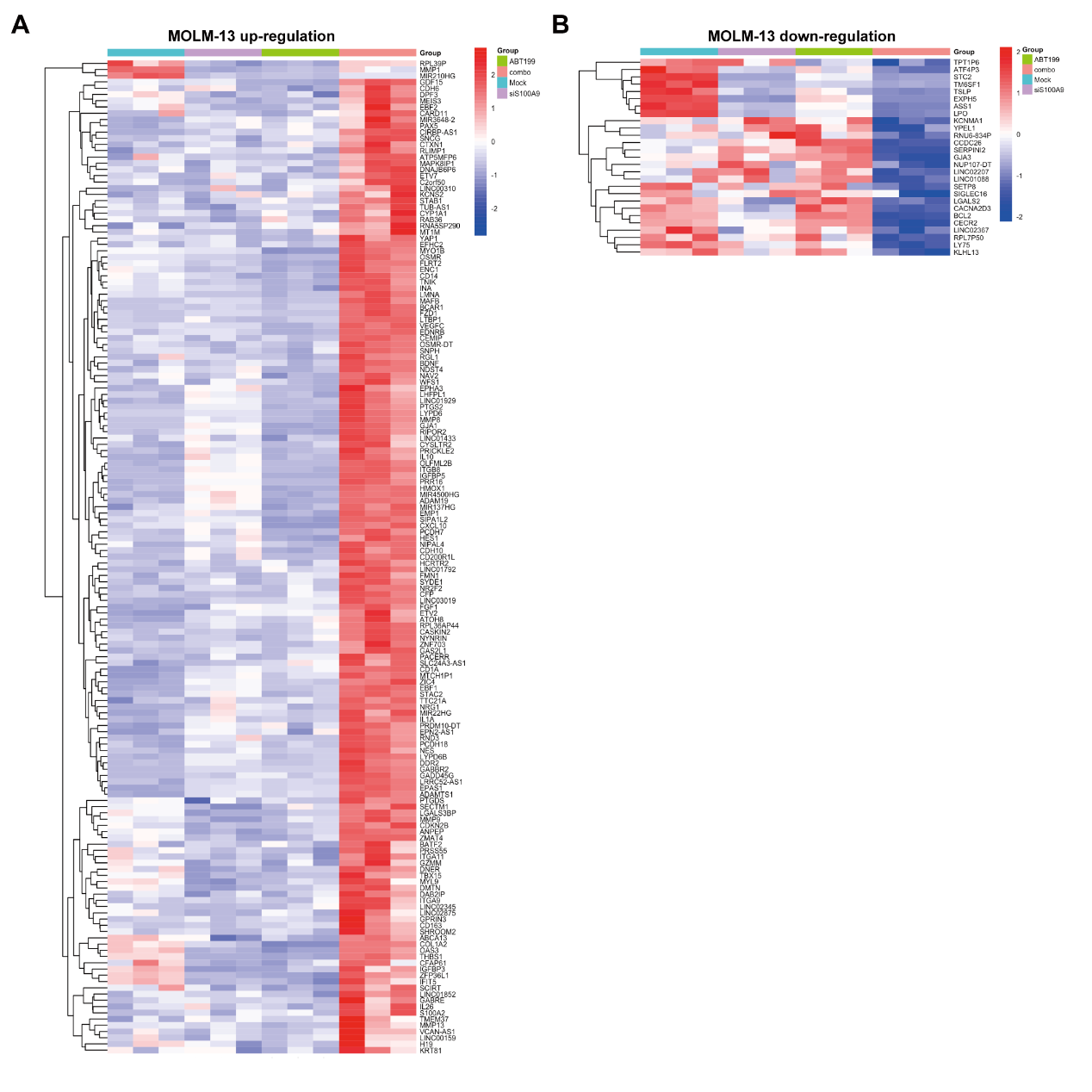


**Supplemental Figure 10.** MOLM-13 cells were treated with 20 nM siS100A9 for 48 hours and afterwards venetoclax (250 nM) was added for an additional 24 hours. Heat map of the intersection genes of venetoclax vs combo and siRNA vs combo of the RNA sequencing data from MOLM-13 cells. Left, up-regulated genes, right, down-regulated genes. (**A**) Up-regulation. (**B**) Down-regulation. Biological triplicate experiments were conducted and represented by 3 columns for each condition.

**
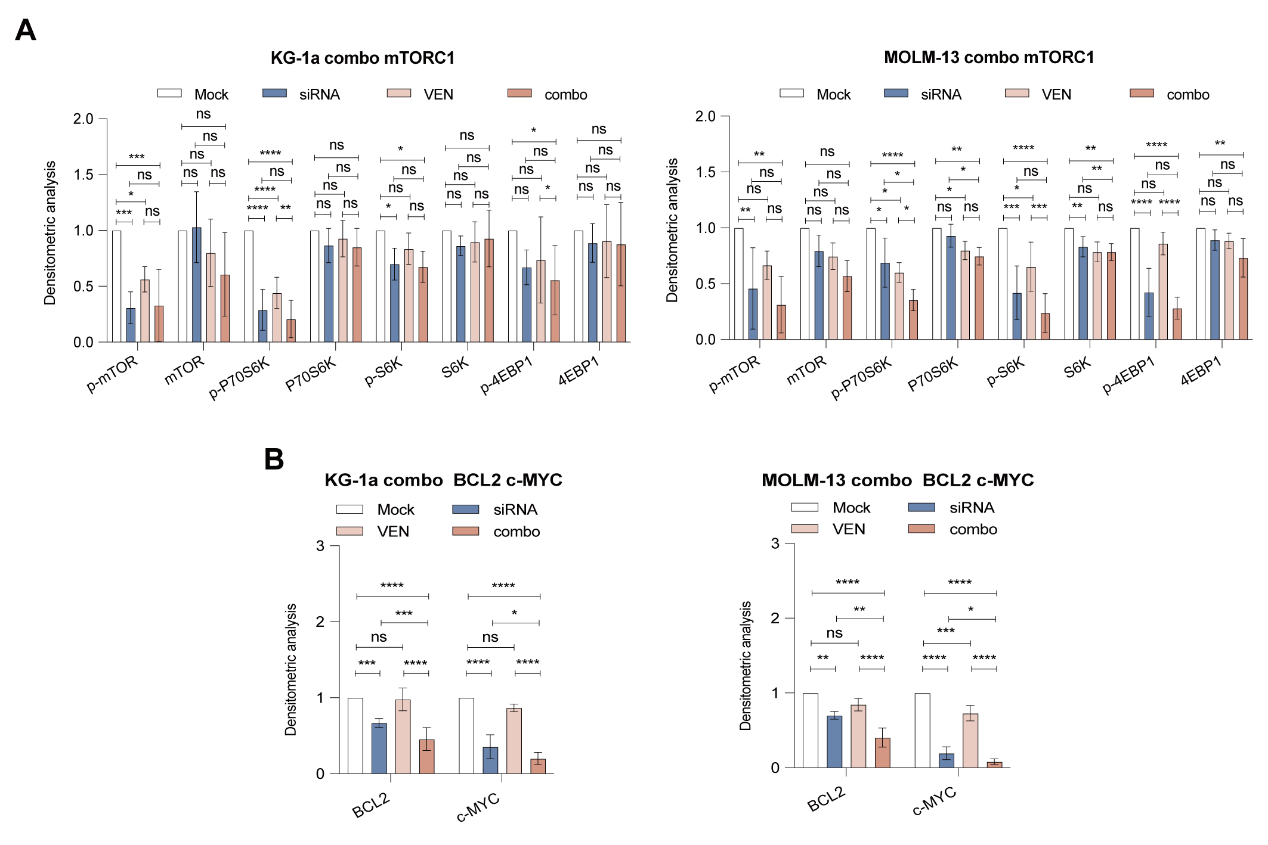
**

**Supplemental Figure 11. Grayscale analyses of western blots in Figure 5.** Human AML cell lines KG-1a and MOLM-13 were treated with 20 nM siS100A9 for 48 hours and afterwards venetoclax (250 nM) was added for an additional 24 hours. Western blot signals were analyzed using ImageJ software. (**p* < 0.05, ***p* < 0.01, ****p* < 0.001, *****p* < 0.0001, One-way ANOVA, Error bars indicate SD).

**
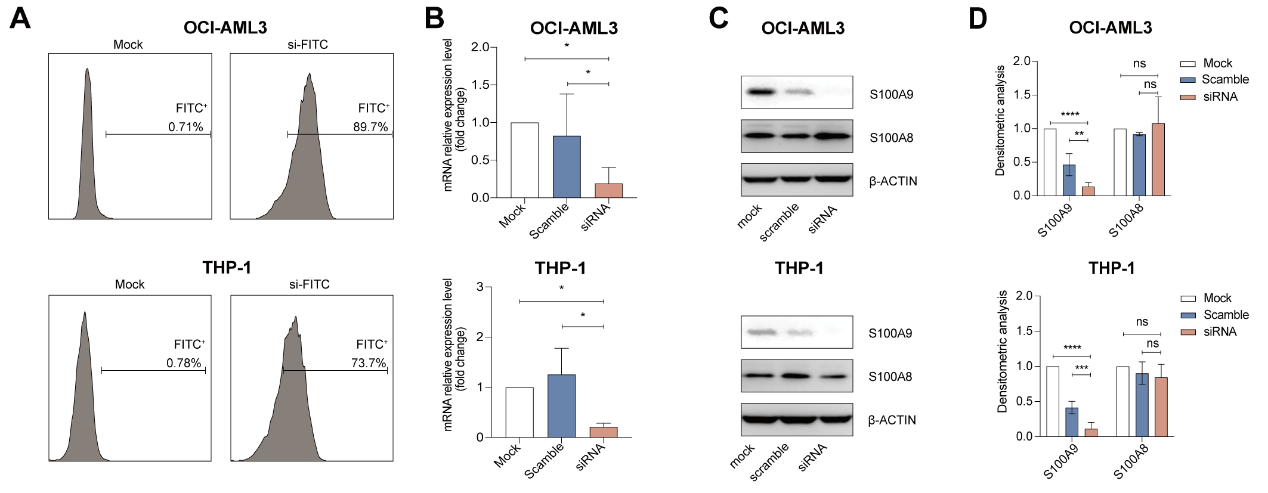
**

**Supplemental Figure 12. Transfection efficiency and validation of S100A9 silencing using S100A9-siRNA in venetoclax-resistant AML cell lines OCI-AML3 and THP-1.** (**A**) OCI-AML3 and THP-1 cells were transfected with 20 nM (OCI-AML3) or 60 nM (THP-1) FITC-labeled siRNA. After 24 hours, cells were analyzed by flow cytometry to assess the transfection efficiency. (n = 3, one representative figure is shown) (**B-C**) OCI-AML3 and THP-1 cells were transfected with 20 nM S100A9-siRNAs (OCI-AML3) or 60 nM S100A9-siRNAs (THP-1). A mock (only lipofectamine) and scramble condition (negative control) were included as controls. S100A9 mRNA (48 hours after transfection) and protein levels (72 hours after transfection) were measured using real-time PCR and western blot respectively. ABL (for real-time PCR) and β-actin (for western blot) were included as internal controls. (**D**) Grayscale analyses of western blots using ImageJ software. (n = 3) (**p* < 0.05, ***p* < 0.01, ****p* < 0.001, *****p < 0.0001*, One-way ANOVA, Error bars indicate SD).

**
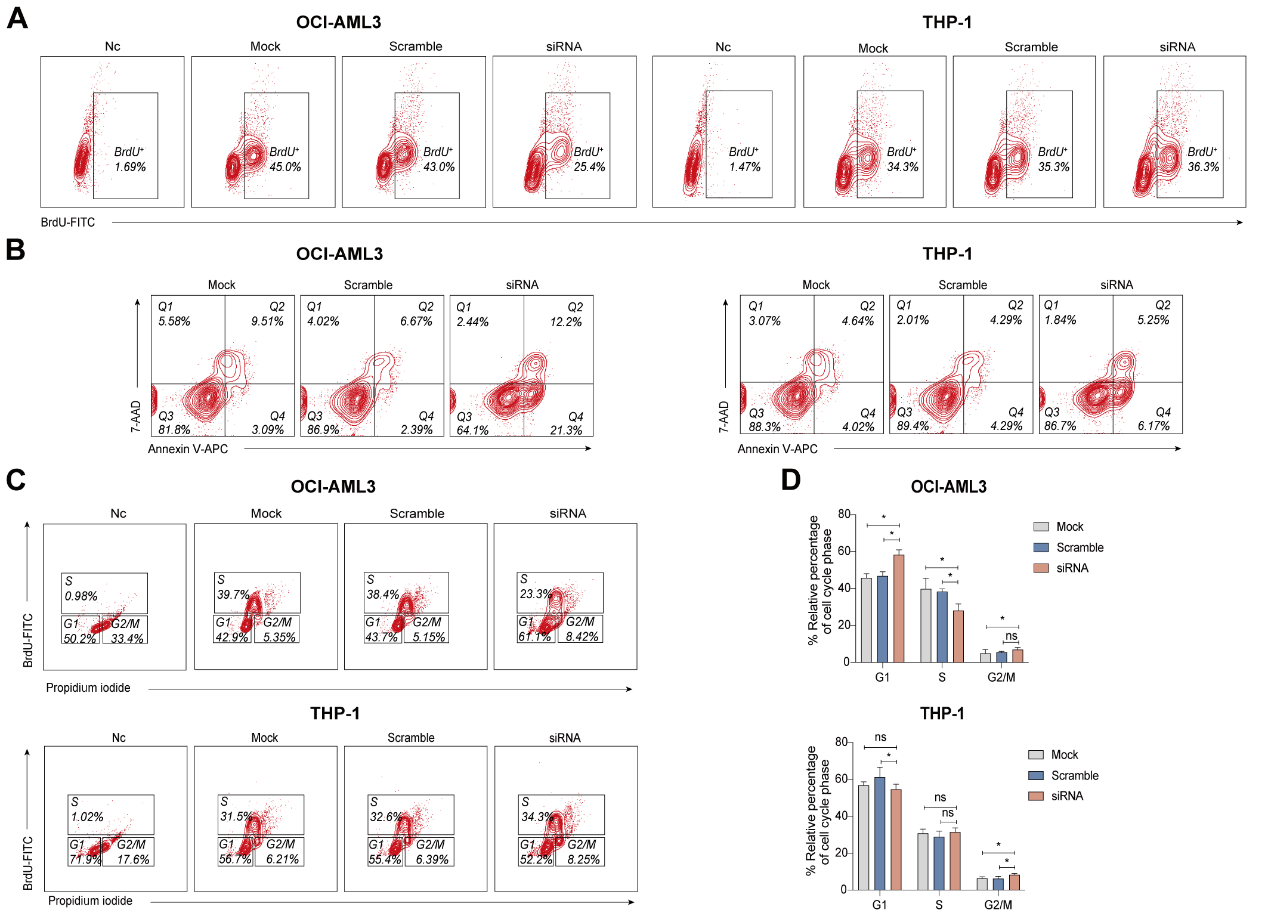
**

**Supplemental Figure 13. Effect of S100A9-siRNA on cell viability, proliferation and apoptosis of venetoclax-resistant AML cell lines.** OCI-AML3 and THP-1 cells were exposed to 20 nM or 60 nM siRNA S100A9 and Lipofectamine 2000 for 48 hours (n = 4). A mock (only lipofectamine) and scramble condition (negative control) were included as controls. (**A**) Cell proliferation was analyzed using a BrdU staining and flow cytometry. Representative figures and the gating strategy are shown. (**B)** Apoptosis was detected by flow cytometry using an Annexin V/7-AAD staining and representative figures (including gating strategy) are shown. (**C, D**) Cells cycle analysis was assessed using a BrdU/Propidium iodide staining. Representative figures and the gating strategy are shown. (**p* < 0.05, One-way ANOVA, Error bars indicate SD).

**
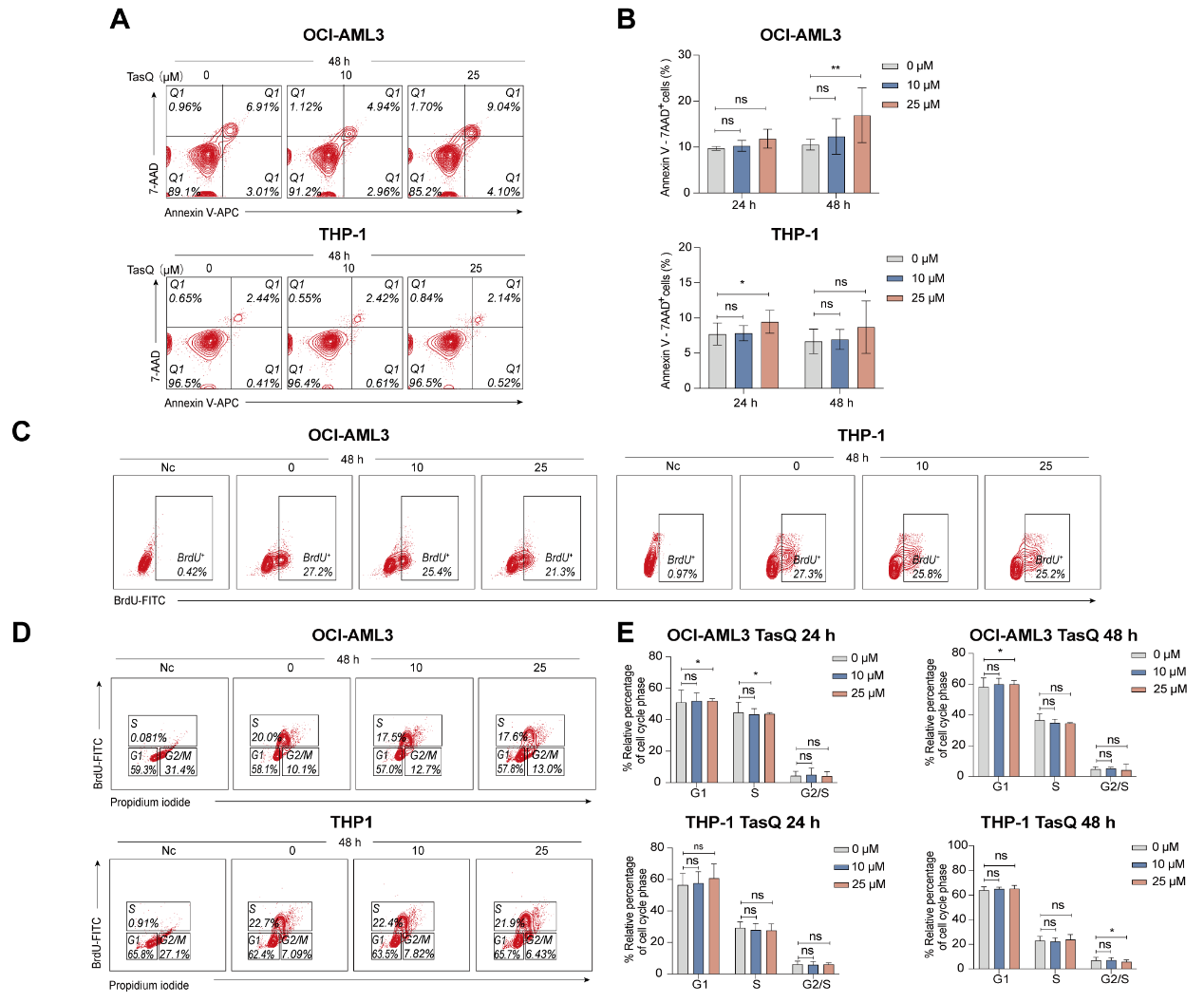
**

**Supplemental Figure 14. Effect of tasquinimod on cell apoptosis and proliferation of venetoclax-resistant AML cell lines.** OCI-AML3 and THP-1 cells were exposed to different concentrations (0, 10, 25 μM) of tasquinimod for 24 and 48 hours (n = 4). (**A, B**) Cell apoptosis was detected by flow cytometry using an AnnexinV/7-AAD staining and representative figures (including gating strategy) are shown. (**C**) Cell proliferation was analyzed using BrdU staining and flow cytometry. Representative figures and the gating strategy are shown. (**D, E**) Cells cycle analysis was assessed using BrdU/ Propidium iodide staining. Representative figures and the gating strategy are shown. (**p* < 0.05, ***p* < 0.01, One-way ANOVA, Error bars indicate SD).

**
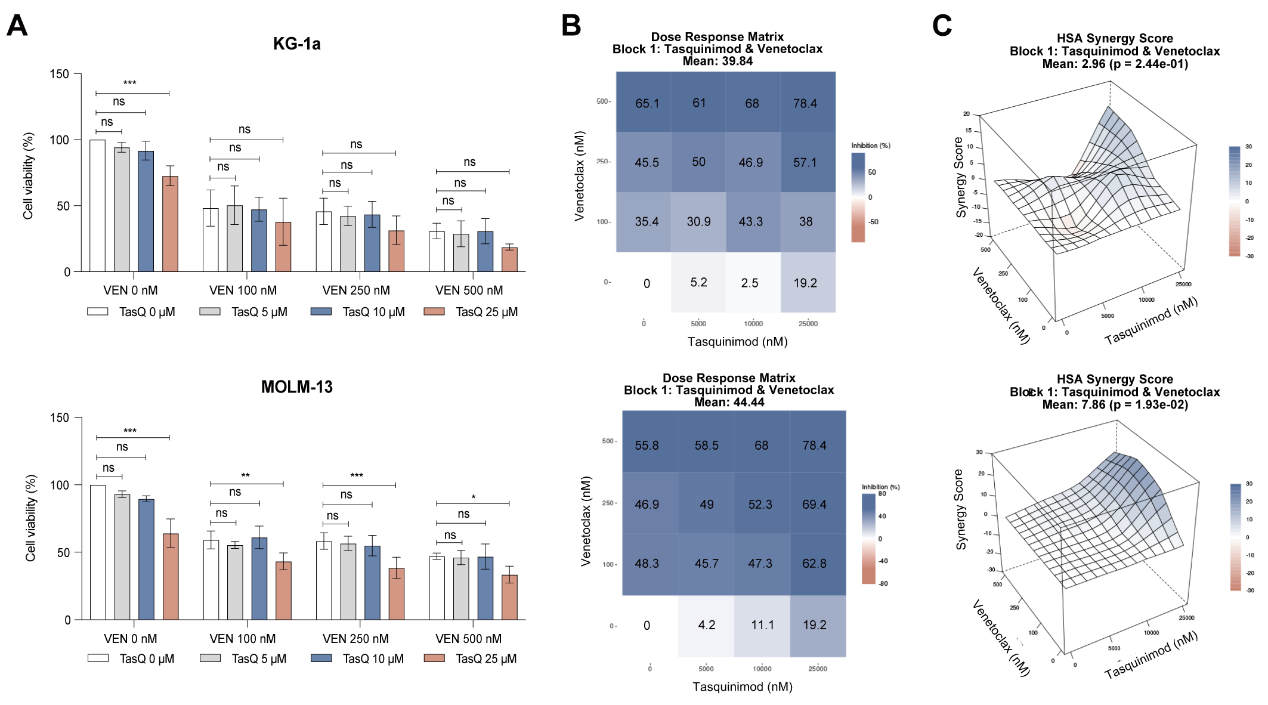
**

**Supplemental Figure 15. The combination effect of tasquinimod and venetoclax on human AML cell lines.** KG-1a and MOLM-13 cell lines were treated with tasquinimod (5, 10, 25 μM) for 24 hours. After 24 hours, venetoclax (50, 250, 500 nM) was added for an additional 24 hours. (**A**) Cell viability was determined by CellTiter-Glo. (**B**) The percentage growth inhibition is shown for all single agents and combinations compared to control. (**C**) The synergy score is calculated using SynergyFinder plus online. The eﬀect of the drug combination (synergism /additive eﬀect /antagonism) was calculated and visualized using SynergyFinder plus software and HSA (Highest single agent) reference model. Blue regions - synergism; white - additive eﬀect; pink – antagonism (n = 4). (**p* < 0.05, ***p* < 0.01, ****p* < 0.001, One-way ANOVA, Error bars indicate SD).


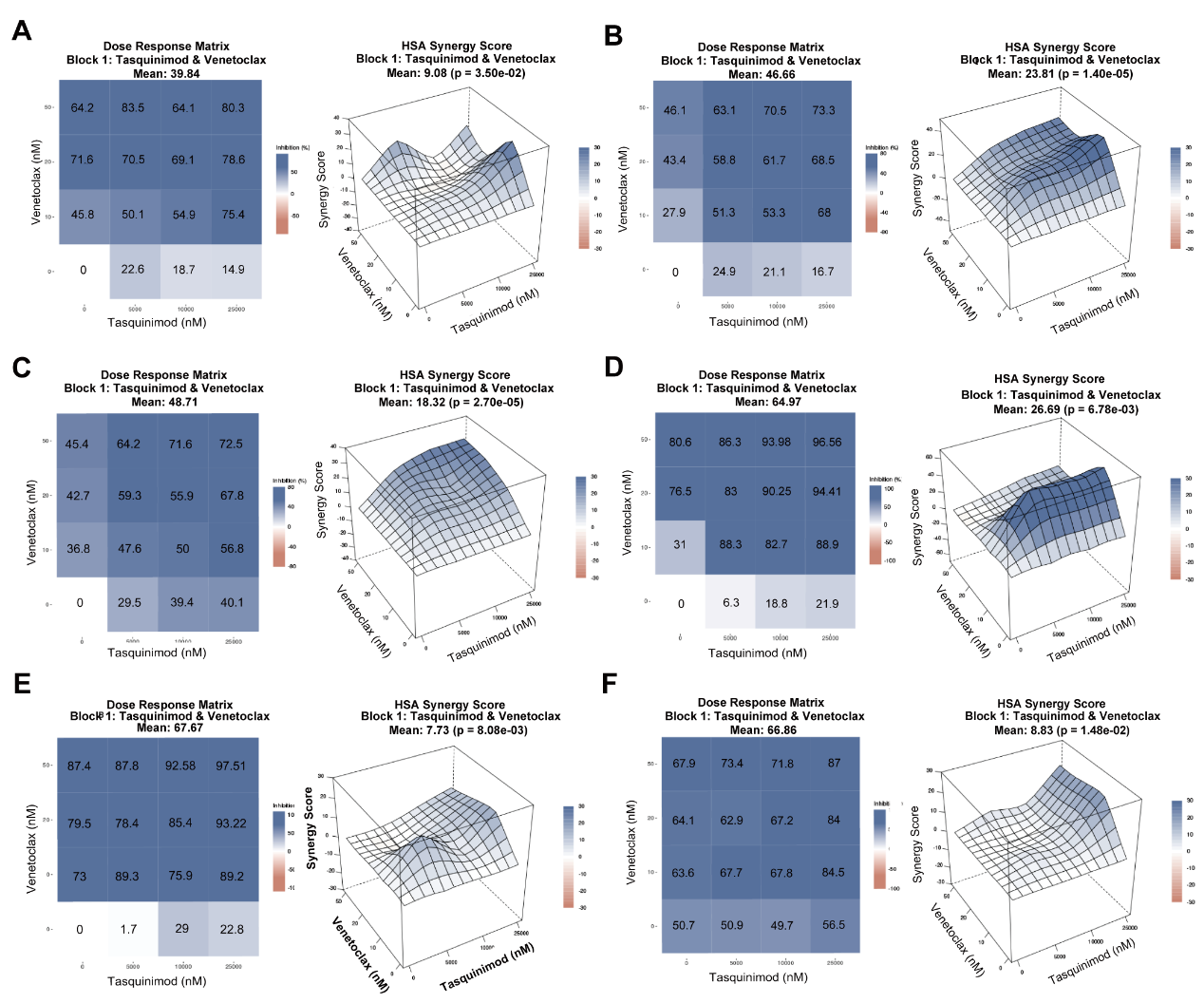


**Supplemental Figure 16. The combination effects between tasquinimod and venetoclax in primary AML samples.** (**A-E**) BMMC and PBMC of AML patients were treated with tasquinimod (5, 10, 25 μM) for 24 hours. After 24 hours, venetoclax (50, 250, 500 nM) was added for an additional 24 hours. Cell viability was determined by CellTiter-Glo for patient 1 (**A**), patient 2 (**B**), patient 3 (**C**), patient 4 (**D**) and patient 5 (**E**). (**F**) Apoptosis was measured by flow cytometry using an AnnexinV/7-AAD staining. On the left side, the percentage growth inhibition is shown for all single agents and combinations compared to control. On the right side, 3D synergy maps are shown to illustrate additive/synergistic effects as calculated and visualized using the SynergyFinder plus software and HSA model. Blue regions - synergism; white - additive eﬀect; pink - antagonism.


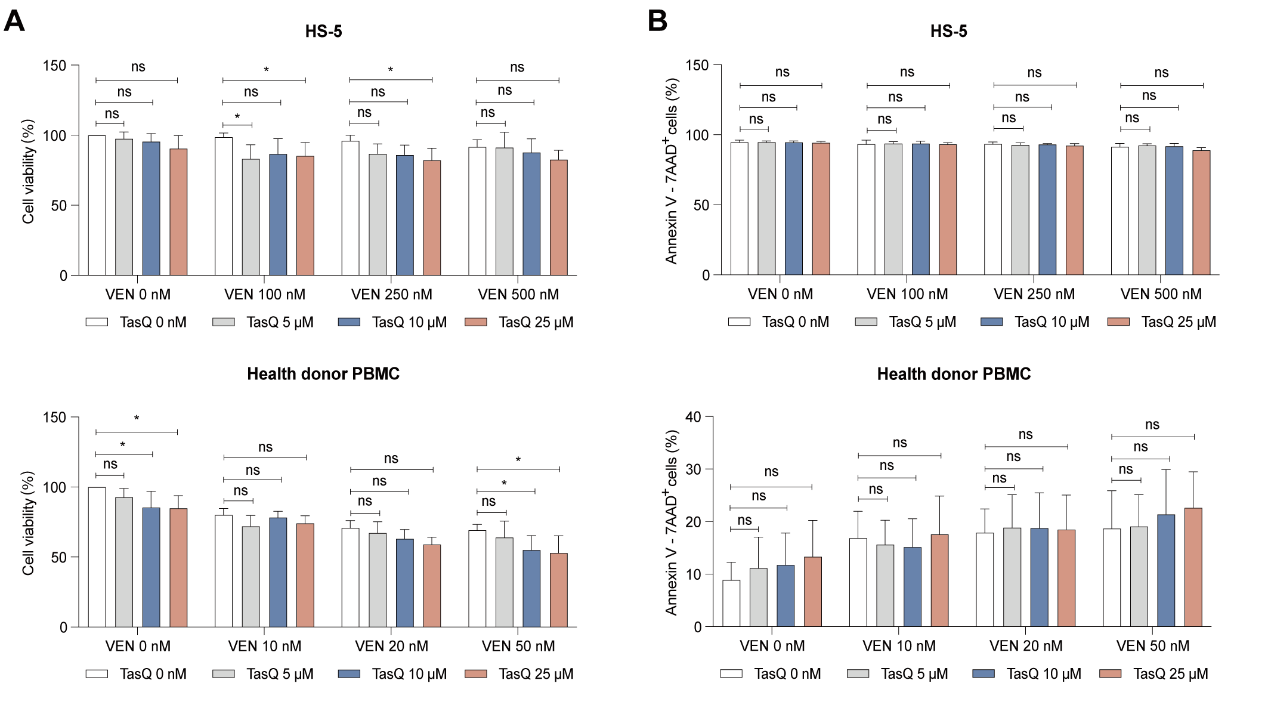


**Supplemental Figure 17.** Effects of venetoclax and tasquinimod on cell viability and apoptosis of HS-5 and healthy PBMC. HS-5 (n = 4) and PBMC (n = 4) were treated with indicated concentrations of venetoclax and tasquinimod for 48h. **(A)** Cell viability was analysed using Cell Titer Glo. **(B)** Apoptosis was analysed AnnexinV/7-AAD staining followed by flow cytometry respectively. (**p* < 0.05, ***p* < 0.01, One-way ANOVA, Error bars indicate SD).
